# Indoleamine 2, 3-Dioxygenase Promotes Aryl Hydrocarbon Receptor-Dependent Differentiation Of Regulatory B Cells in Lung Cancer

**DOI:** 10.3389/fimmu.2021.747780

**Published:** 2021-11-19

**Authors:** Sultan Tousif, Yong Wang, Joshua Jackson, Kenneth P. Hough, John G. Strenkowski, Mohammad Athar, Victor J. Thannickal, Robert H. McCusker, Selvarangan Ponnazhagan, Jessy S. Deshane

**Affiliations:** ^1^ Department of Medicine, University of Alabama at Birmingham, Birmingham, AL, United States; ^2^ Department of Dermatology, University of Alabama at Birmingham, Birmingham, AL, United States; ^3^ Department of Animal Sciences, University of Illinois at Urbana Champaign, Urbana, IL, United States; ^4^ Department of Pathology, University of Alabama at Birmingham, Birmingham, AL, United States

**Keywords:** IDO, L-Kynurenine, Breg cells, MDSC (myeloid-derived suppressor cells), TME (tumor microenvironment), immunosuppression, lung cancer, aryl hydrocarbon receptor (AhR)

## Abstract

Regulatory B cells (Breg) are IL-10 producing subsets of B cells that contribute to immunosuppression in the tumor microenvironment (TME). Breg are elevated in patients with lung cancer; however, the mechanisms underlying Breg development and their function in lung cancer have not been adequately elucidated. Herein, we report a novel role for Indoleamine 2, 3- dioxygenase (IDO), a metabolic enzyme that degrades tryptophan (Trp) and the Trp metabolite L-kynurenine (L-Kyn) in the regulation of Breg differentiation in the lung TME. Using a syngeneic mouse model of lung cancer, we report that Breg frequencies significantly increased during tumor progression in the lung TME and secondary lymphoid organs, while Breg were reduced in tumor-bearing IDO deficient mice (IDO^-/-^). Trp metabolite L-Kyn promoted Breg differentiation *in-vitro* in an aryl hydrocarbon receptor (AhR), toll-like receptor-4-myeloid differentiation primary response 88, (TLR4-MyD88) dependent manner. Importantly, using mouse models with conditional deletion of IDO in myeloid-lineage cells, we identified a significant role for immunosuppressive myeloid-derived suppressor cell (MDSC)-associated IDO in modulating *in-vivo* and *ex-vivo* differentiation of Breg. Our studies thus identify Trp metabolism as a therapeutic target to modulate regulatory B cell function during lung cancer progression.

## Introduction

Lung Cancer is the leading cause of cancer-associated morbidity and mortality among men and women, claiming ~1.3 million deaths annually worldwide (1–3). The predominant form of lung cancer is non-small-cell lung carcinoma (NSCLC), which accounts for about 85 percent of all lung cancers. Immunotherapy strategies have provided only limited improvement in 5-year survival for NSCLC.

The tumor microenvironment (TME) plays a dynamic role in regulating immune responses during the progression of lung cancer (4, 5). Tumors infiltrating immune cells not only inhibit but also promote tumor growth (6–8). Tumor promoting myeloid-derived suppressor cells (MDSCs) and regulatory T cells (Treg) are known contributors of immunosuppression in the TME (9, 10). But recent studies have emphasized a regulatory function also for subsets of B cells called regulatory B cells (Breg) in inhibiting anti-tumor immune responses (11–16). Increased frequency of circulating Breg was recently reported in patients with pathological stage of lung cancer (17). Among the widely described immunosuppressive characteristics of Breg is the production of the potent anti-inflammatory cytokine IL-10 (18, 19). IL-10 producing B cell subsets (Breg) that are CD1d high were first identified by Mizuguchi et al. in 2002 in the study of chronic intestinal inflammation (20). Breg was shown to regulate autoimmunity in murine models of systemic lupus erythematosus (SLE), type 1 diabetes, arthritis and chronic intestinal inflammation (21–25). Previous studies have also reported that Breg suppress anti-tumor T cell responses through induction of Treg (10).

The significance of Bregs in anti-tumor immunity became clear when improved therapeutic efficacy was observed in lung tumor-bearing B cell-deficient mice following treatment with a combination of chemotherapy and IL-15 (26). A significant increase in tumor burden was also reported following the adoptive transfer of Breg in a murine model of lymphoma with anti-CD20 antibody treatment (27, 28). Moreover, Breg contribute to immunosuppression in pulmonary metastasis during breast cancer progression (29). However, the underlying mechanisms of Breg development during the progression of lung tumors still remain mostly unknown.

Lineage-specific transcription factors have so far not been identified for Breg, suggesting that Breg are reactive rather than lineage specific. It is well known that immature B cells, mature B cells and plasmablasts may differentiate into IL-10 producing Breg through activation of Toll-like receptor (TLR) and CD40 (30, 31). Immunosuppressive properties of B cells, especially the production of IL-10 in experimental autoimmune encephalomyelitis (EAE) mice model is dependent on toll-like receptor-2 (TLR-2), toll-like receptor-4 (TLR-4)and myeloid differentiation primary response 88 (MyD88) signaling (32). Moreover, the development of Breg in thymoma patients with myasthenia gravis was regulated by TLR9/MyD88/NF- kappa B signaling pathway (33). These studies collectively support the hypothesis that Breg differentiation occurs in response to positive stimuli rather than by regulation *via* a specific lineage factor. Thus, investigating novel mechanisms of regulation of Breg will lead to the identification of promising targets for cancer therapeutics.

Recently we showed that while MDSCs inhibit B cell responses during lung tumor progression, with increased infiltration of Breg in the lung (34). MDSCs are heterogeneous and immature myeloid cells that are established drivers of immune suppression during tumor progression (35–38). While MDSCs are known to induce Treg expansion and activation (39, 40), recent studies suggest that MDSCs may promote Treg through Breg (15). In our studies, we also identified that MDSCs are major contributors of Indoleamine 2, 3-dioxygenase (IDO) activity in the lung TME (41). IDO is a rate-limiting tryptophan (Trp) metabolizing enzyme that is expressed in MDSCs and tumor cells. IDO has emerged as a potent immunosuppressive pathway for MDSCs for suppression of anti-tumor CD8^+^ T cells and the expansion of Treg (42–45). Trp metabolite L-Kynurenine (L-Kyn), a well-known endogenous ligand of aryl hydrocarbon receptor (AhR), activates AhR to induce immunosuppression through Treg expansion (46). Furthermore, L-Kyn promotes tumor growth by inhibiting anti-tumor immune responses *via* AhR in an autocrine/paracrine fashion (47). A potential role for Trp metabolism as a regulator of Breg differentiation and function has not been investigated yet.

In this study, we provide direct evidence for Trp-metabolite L-Kyn in modulating differentiation of Breg *in vitro*. Our studies with a syngeneic model of lung cancer show impaired infiltration of Breg in TME and spleens of IDO deficient (IDO^-/-^) mice suggesting an important role for IDO pathway in Breg infiltration and differentiation. Further, using mice with conditional deletion of IDO in myeloid lineage cells, we provide evidence that MDSC-associated IDO contribute significantly to Breg infiltration during lung cancer progression. Additionally, *in-vivo* and *ex-vivo* studies with AhR deficient mice (AhR^-/-^) indicate a potential role for AhR in Breg differentiation and corroborate our hypothesis that L-Kyn induced differentiation of Breg precursors is AhR independent whereas IL-10 producing Breg require AhR signaling. In addition, our *ex-vivo* data generated from TLR-4, MyD88, TLR-2, and TLR-2, 4 deficient B cells suggest a crucial role of TLR-4-MyD88 signaling in L-Kyn induced Breg differentiation. Collectively, our present study reveals a novel role of IDO and the Trp metabolite L-Kyn in Breg differentiation during the progression of lung cancer.

## Material and Methods

### Cell Culture and Mice

Lewis lung carcinoma cells (LLCs) (American Type Culture Collection (ATCC) (Manassas, VA)) were propagated in Dulbecco’s Modified Eagle Medium (DMEM) with supplementation of 10% fetal bovine serum (FBS), 2 mM L-glutamine, 1 mM sodium pyruvate, 10 μg/ml penicillin-streptomycin and 0.1 mM non-essential amino acids (Life Technologies, Waltham, MA). LLCs (1 x10^6^ LLC cells per mouse in 100 μl PBS) at 70-80% confluency were injected *via* intracardiac (i.c.) or tail vein (i.v.) route into. C57BL/6 and B6129-Ido1/J (IDO^-/-^) mice (Jackson Laboratories (Bar Harbor, ME). Tumor weights at early time points in IDO deficient mice are not accurately measurable and hence these measurements were made 9 days post tumor implantation as we have described before (41). AhR^-/-^ mice were obtained from a UAB colony maintained by John F. Kearney. TLR-2^-/-^, TLR-4^-/-^ TLR-2, 4^-/-^, and MyD88^-/-^ mice were obtained from UAB colony maintained by Dr. Suzanne M. Michalek.

### IDO f MCre Mice

MCre and IDO f MCre mice were obtained from Robert H. McCusker, University of Illinois at Urbana-Champaign. Ido1 floxed mice (Ido1fl) were generated by inserting a 75bp loxP cassette 5’ of the second exon of the reference gene, NM_008324.2, by Ingenous targeting laboratory (Ronkonkuma, NY). A second loxP site was inserted 3’ of the fourth exon. Targeting was performed with C57BL/6 embryonic stem cells and microinjected into Balb/c blastocysts. Chimeras with a high percentage black coat color were mated to C57BL/6 FLP mice to remove the Neo cassette. Cre recombinase excises a 2.37 kb region of the Ido1 gene. Ido1M-Cre mice were generated by crossbreeding Ido1fl mice to M-Cre mice (B6.129P2-Lyz2tm1(cre)Ifo/J; JAX 004781) to target Ido1 inactivation in myeloid-derived cells. Mice were bred to homozygosity for ease of colony propagation. Both male and female mice of 6-8 weeks old were used for *in-vitro* and *in-vivo* experiments. All mice used in this study were bred and maintained in pathogen-free animal facility at Research Support Building, UAB at Birmingham, AL, and handled in accordance with the Guidelines for Animal Experiments at the University of Alabama at Birmingham, AL, USA.

### Antibodies and Reagents

We used the following antibodies and reagents: anti-CD19 (clone: eBio1D3)-PE-Cy7, anti-CD5 (clone: 53-7.3)-APC, anti-CD1d (clone: 1B1)-eFluor 450, anti-IL-10 (clone: JES5-16E3)-PE, anti-Gr-1 (clone: RB6-8C5)-PE-Cy5, anti-CD11b (clone: M1/70)-APC-Cy7)-PE, anti-TCRαβ (clone: 145-2C11)-PE-Cy5,anti-IgM (clone: II/41)-FITC, anti-IgD (clone: 11-26C)-PerCPeFluor710, anti-B220 (clone: RA3-6B2)-APC-eFluor780, anti-CD21 (clone: eBio4E3)-FITC, anti-CD43 (clone: eBioR2/60)-PE, anti-CD23 (clone: B3B4)-PE, anti-CD93 (clone: AA4.1)-APC, anti-CD24 (clone: M1/69)-APC, and anti-IgM (clone II/41)-eFluor 450. All the antibodies were purchased from ebiosciences, USA. Anti-CD3 (clone: 145-2C11)-APC-Cy7 and the purified rat anti-mouse CD16/CD32 (Mouse BD Fc Block™) (clone#2.4G2) was from BD Pharmingen, USA. Polyclonal AhR antibody (cat# BML-SA210-0025) was obtained from Enzo Life Sciences, USA. Histone H4 Antibody (cat# SC25260) and GAPDH Antibody (cat # SC365062) from Santa Cruz Biotechnology, USA. Anti-Rabbit IgG HRP conjugate (cat # W401B) and Anti-mouse IgG HRP conjugate (cat # W402B) from Promega, USA. L-Kyn (cat# K8625), Lipopolysaccharide (LPS) (cat# L4391), AhR antagonist CH-223191 (cat# C8124) and 1-Methyl-D-tryptophan (D-1MT) (cat# 452483) from Sigma-Aldrich. Recombinant murine- rIFN-ϒ (cat# 315-05) from PeproTech and CellTrace CFSE (cat# C34554) from invitrogen USA. Easy-Sep™ Mouse B Cell Isolation Kit (cat# 19854) from Stem Cell Technology, USA, Cell fractionation kit-Standard (cat# ab109719) from abcam, USA, Pierce BCA Protein Assay Kit (cat# 23225) from Thermo Scientific, USA, Pure-Link™ RNA Mini Kit (cat# 12183018A) from Invitrogen, USA, Prime script 1^st^ strand cDNA synthesis kit (cat# 6110A) from TaKaRa Biotechnology, USA, VeriQuest SYBR Green qPCR master mix from Affymetrix, USA and Mouse IL-10 ELISA kit (cat# 88-7105-22) from ebioscience, USA.

### Flow Cytometry Analyses

Spleens were isolated from mice and macerated in RPMI 1640 (Gibco, Invitrogen, UK) supplemented with 10% FBS to prepare a single-cell suspension. Isolated tumor tissues were first macerated in serum-free Iscove’s Dulbecco’s Modified Eagle Medium (IDMEM) (Corning) and then digested in serum-free IDMEM contained with 2 mg/ml of collagenase B (Roche) and 0.02 mg/ml of deoxyribonuclease from bovine pancreas (Sigma) at 37°C for 30 min. After digestion, suspensions were neutralized with sterile IDMEM supplemented with 10% FBS filtered through 0.45 mm strainers. Red blood cells (RBCs) were lysed with ACK lysis buffer (Quality Biological, Inc.) incubated at room temperature for 1 minute and washed with 10% RPMI 1640. The cells were counted, and 0.5-1×10^6^ cells were used for surface staining post Fc blocking (1μg/ml) in 3% BSA or FBS for 30 minutes over ice. For intracellular IL-10 staining 0.5-1×10^6^ cells were cultured per well in 96-well plates (Nunc, USA) then incubated for 5hrs in stimulating RPMI media containing Phorbol 12-myristate 13-acetate, or PMA, (0.1 mg/ml, Sigma), ionomycin (1 mg/ml, Sigma), LPS (10 µg/ml, Sigma) and a 1:1000 dilution of Golgistop/Golgiplug (BD Biosciences). Following stimulation, cells were first surface stained for 30 minutes at ice. After washing with PBS cells were fixed and permeabilized using the BD cytofix/cytoperm kit (BD Biosciences) for 30 minutes at 4°C then washed again with BD perm wash kit (BD Biosciences) and stained with intracellular fluorescently labeled anti-IL-10 antibodies. Fluorescence intensity of fluorochrome-labeled cells was measured by flow cytometry (BD LSR-II) while required sorting was done by BD FACS Aria III cytometer (UAB Flow Cytometry Core Facility). FACS Diva was used for acquiring the cells, and final data analysis was performed by Flow Jo (Tree star, USA). We included the whole nuclear cell population, including the tumor cells (CD45^neg^) for the analyses of % total Breg.

### B Cells Purification and *In-Vitro* Breg Differentiation

Spleens or Bone marrow (BM) were isolated from mice and macerated in RPMI 1640 (Gibco, Invitrogen, UK) supplemented with 10% FBS to prepare a single-cell suspension. Red blood cells (RBCs) were lysed with ACK lysis buffer (Quality Biological, Inc.), then washed with 10% RPMI 1640. Cells were rewashed again in 1x PBS, and B cells were purified following manufacturer’s protocol. Briefly, 1x10^8^/ml splenocytes were resuspended in RoboSep buffer (Stem cell technology, USA) followed by the addition of rat serum (50µl/ml) (Stem cell technology, USA) to the sample. Samples were transferred then to 5ml polystyrene round-bottom tube and B cell isolation Antibodies cocktail (50µl/ml) was added and mixed by pipetting, then incubated for 10 minutes at room temperature (RT). Rapid sphere (75µl/ml) (Stem cell technology, USA) was then added to the sample after vortexing it for 30 sec then mixed and incubated for 2.5 minutes at RT. RoboSep buffer was topped up to sample to make total volume 2.5ml and pipetted gently. Sample tubes were then placed in magnet (Easy Sep Magnet,cat# 18000, stem cell technology, USA) and incubated for 2.5 minutes at RT. Magnet was picked up and inverted the magnet tube pouring off the enriched cell suspension into a new tube. Purified B cells were washed twice with 10% RPMI 1640 and counted. B cells were disaggregated and stained with CFSE if needed and then 5 x10^5^ cells were seeded in 200 µL per well in a 96 round bottom well plate with LPS (10μg/ml), increasing concentrations of L-Kyn (50µM and 100μM) and LPS + L-Kyn + CH 223191(10μM) an aryl hydrocarbon receptor antagonist (AhRA) for 72 hrs. Bregs were identified as CD19^+^CD5^+^CD1d^hi^IL-10^+^ by flow cytometry.

### *In-Vitro* Co-Culture

For the *in-vitro* co-culture of B cells and MDSCs, CD19^+^ B cells were purified from splenocytes of naïve mice and co-cultured in a 5:1 ratio with MDSCs (CD11b^+^Gr-1^+^) sorted from the spleens and tumors of tumor-bearing mice and incubated for 72 hrs. For the *in-vitro* co-culture of B and T cells, T cells (CD3^+^TCRαβ^+^) were sorted from splenocytes of naïve mice and co-cultured in 1:1 ratio with purified B cells and incubated for 72 hrs. Bregs were identified in the co-culture as CD19^+^CD5^+^CD1d^hi^IL-10^+^ by flow cytometry.

### Cell Fractionation and Immunoblotting

Cytoplasmic and nucleoplasmic fractions were extracted from cell pellets collected from *ex-vivo* experiments following stimulation with LPS and LPS+L-Kyn using protocols provided with the Cell fractionation kit-Standard (cat# ab109719, Abcam, USA). Protein concentrations were measured using Pierce BCA Protein Assay Kit (cat# 23225, Thermo Scientific, USA). 20-40µg of the cytoplasmic and nucleoplasmic fractions were used for western blot analysis to detect AhR expression on 10% SDS-PAGE gels. Gels were transferred overnight on to 0.45 μm Immobilon-P PVDF membranes (EMD Millipore). Blots were blocked in 5% non-fat dry milk (Lab Scientific) for 1 hour and incubated with primary antibody (manufacturer’s recommended concentrations) overnight at 4°C and then incubated in secondary anti-rabbit or anti-mouse HRP-conjugated antibodies (Promega, City, State), and protein bands detected using chemiluminescence (ECL, Santa Cruz Biotechnology).

### RNA Isolation and qRT-PCR

Total RNA was isolated from cell pellets collected from *ex-vivo* experiments following stimulation with LPS, LPS+L-Kyn, LPS+L-Kyn+AhRA, and LPS+AhRA using Pure-Link™ RNA Mini Kit (cat# 12183018A, Invitrogen, USA) and by following manufacturer’s recommendations. cDNA was made using Prime script 1^st^ strand cDNA synthesis kit (cat# 6110A, TaKaRa Biotechnology, USA). Reverse transcription was conducted with Veri Quest SYBR Green qPCR master mix (Affymetrix, USA). The primer sequences used were as follows: AhR (forward-AGGCCAGGACCAGTGTAGAG, reverse-CTTGGATAGTGGAGGAAGCA), Cyp1A1 (forward-TGGATGCCTTCAAGGACTTG, reverse- CAGCTTCCTGTCCTGACAAT) and RPS-18 was selected as reference or housekeeping genes (forward-GAACTCACGGAGGATGAGGT, reverse-TCTAGACCGTTGGCCAGAAC).

### *In-Vitro* B Cell Proliferation Assays

Splenocytes or sorted CD19^+^B220^+^ B cells from tumor-bearing WT or IDO deficient mice were labeled with CFSE (Molecular Probes, Eugen, OR) and culture for 4 days with 20 ug/ml LPS (Sigma-Aldrich, St. Louis, MO) and 10 ng/ml IL-4 (PeproTech, Rocky Hills, NJ). The percentages of CD19^+^CFSE^low^ cells (proliferating cells) were determined by FACS analysis.

### Quantitation of IL-10 and IgG by ELISA

The mouse sera were collected from naive and tumor-bearing mice. The levels of IL-10 were determined by ELISA kits from ebioecience in sera following the manufacturer protocol. The supernatants from splenocytes or purified B cells from tumor-bearing WT or IDO deficient mice were collected on day 4 of culture. The levels of IgG were determined by ELISA kit from Life Technologies (Grand Island, NY) in the supernatants following the instruction of the manufacturer.

### Statistical Analyses

Statistical analyses were performed using Graph pad prism 5 software (La Jolla, CA, USA) and values were presented as mean ± SD unless indicated otherwise in the figure legend. Significant differences between the group means were determined by One-way ANOVA with Tukey’s multiple comparisons test for multiple groups. Comparisons between two groups were evaluated by an unpaired parametric student’s t-test. A value of p<0.05 was considered statistically significant.

## Results

### Tumors and MDSC-Mediated IDO Promotes Breg Differentiation in Lung Cancer

**Figure 1:**
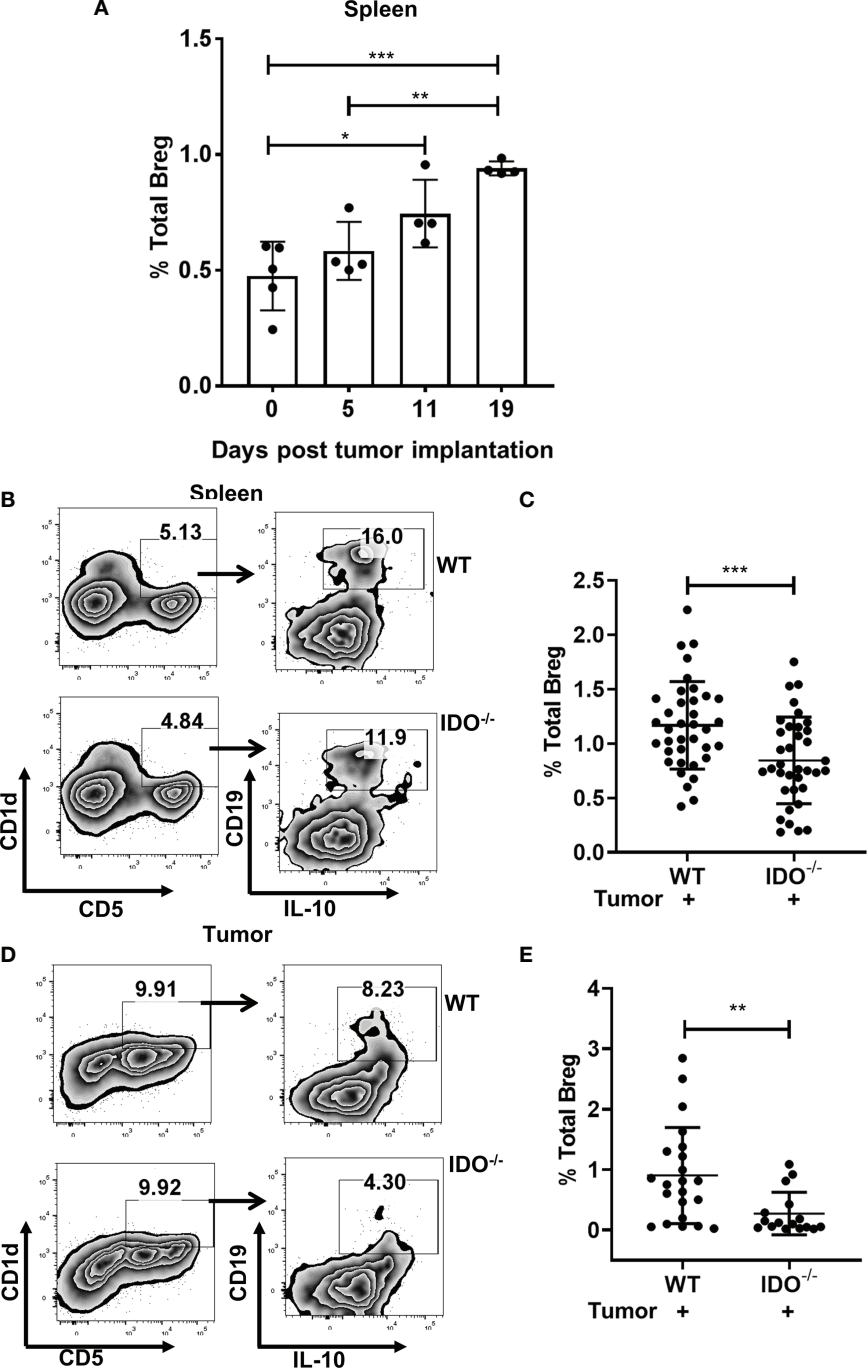
IDO deficiency reduces Breg frequencies in lung cancer. **(A)** Time kinetics of infiltration of Bregs (CD19^+^CD5^+^CD1d^hi^IL-10^+^) during lung cancer progression in tumor-bearing wild type (WT) on Day 5,11 and 19 post i.v. injection of LLC compared to naïve WT mice (n = 4-5 mice per group). **(B)** Representative figure of FACS analyses of splenic tissue confirmed the reduction of Breg (CD19^+^CD5^+^CD1d^hi^IL-10^+^) in tumor-bearing IDO^-/-^ on day 9 post tumor implant. **(C)** Total percentage of Bregs (CD19^+^CD5^+^CD1d^hi^IL-10^+^) in spleens of tumor-bearing WT and IDO^-/-^ mice on Day 9 post tumor implant (n = 36-37 mice per group). **(D)** Representative figure of FACS analyses of lung tumor tissue confirmed reduction of Breg (CD19^+^CD5^+^CD1d^hi^IL-10^+^) in tumor-bearing IDO^-/-^ on day 9 post tumor implant. **(E)** Total percentage of Breg (CD19^+^CD5^+^CD1d^hi^IL-10^+^) in lung tumors of WT and IDO^-/-^ mice on Day 9 post tumor implant (n = 16-22 mice per group). *p < 0.05, **p < 0.001, ***p < 0.0001.

We first investigated if Breg play an important role in the progression of lung cancer. Using a syngeneic murine model of lung cancer with intravenous implant LLCs, we first studied the kinetics of infiltration of CD19^+^CD1d^hi^CD5^+^IL-10^+^ Bregs which significantly increased in a time-dependent manner **(** **Figure 1A** **).** We and others have previously reported that immunosuppressive MDSCs are important drivers of tumor growth in this tumor model (41, 45). Additionally, we have delineated using tumor implanted IDO deficient mice, a critical role for MDSC-associated IDO in dampening anti-tumor T cell responses (42). Recent evidence also suggests that MDSC-mediated regulatory T cell induction is through Bregs (48, 49). Therefore, we first evaluated a potential role for IDO in Breg infiltration during tumor progression using our syngeneic lung cancer model (intracardiac, i.c) in B6129-Ido1/J (IDO^-/-^) and wild type C57BL/6 (WT) control mice. Significantly reduced Breg frequency was observed in spleens and tumors of IDO^-/-^ compared to WT control mice at 9 days following *in-vivo* establishment of lung tumors **(** **Figures 1B–E** and **Supplementary Figure 1A** **)**.

**Figure 2:**
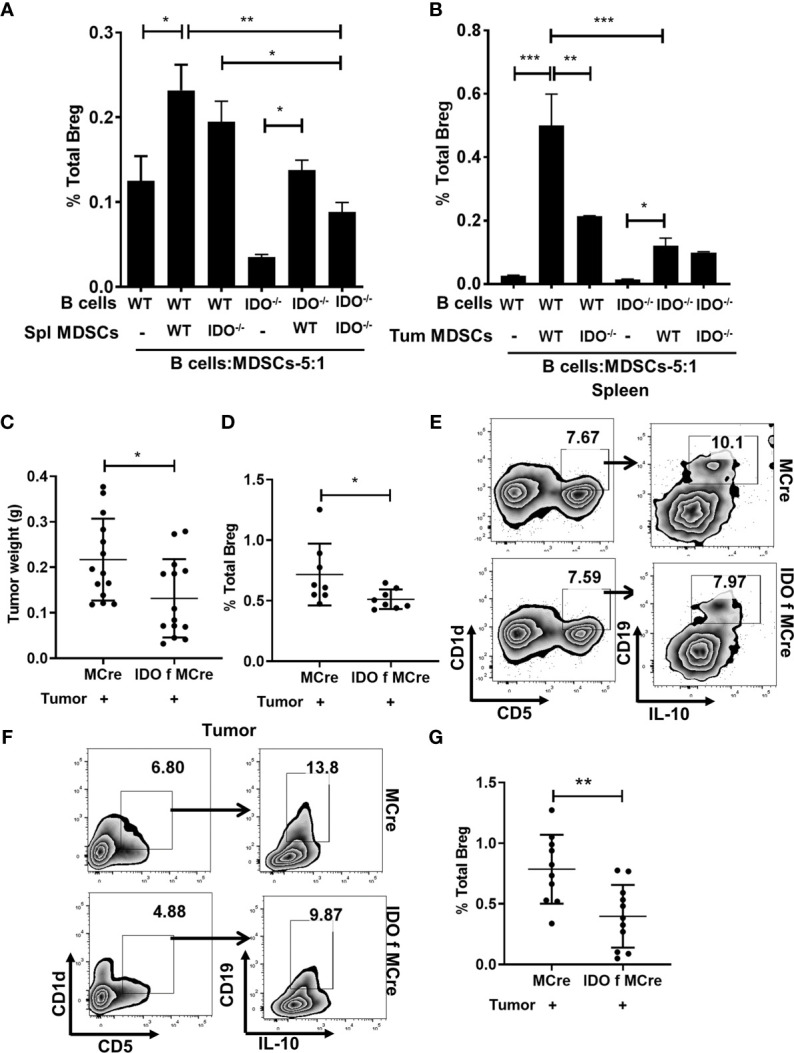
MDSCs expressing IDO promotes Breg differentiation. **(A)** CD19^+^ B cells were purified from splenocytes of WT and IDO^-/-^ naïve mice and co-cultured in 5:1 ratio with MDSCs sorted from the spleen of tumor-bearing WT and IDO^-/-^ and incubated for 72 hrs. Bregs were identified in the co-culture as CD19^+^CD5^+^CD1d^hi^IL-10^+^ by flow cytometry. Data shown here are representative of three independent experiments with three replicates for each group and represent the mean ± STDEV values. **(B)** CD19^+^ B cells were purified from splenocytes of WT and IDO^-/-^ naïve mice and co-cultured in 5:1 ratio with MDSCs sorted from tumor of WT and IDO^-/-^ and incubated for 72 hrs. Bregs were identified in the co-culture as CD19^+^CD5^+^CD1d^hi^IL-10^+^ by flow cytometry. Data shown here are representative of three independent experiments with three replicates for each group and represent the mean ± STDEV values. **(C)** Conditional deletion of IDO in myeloid cells (IDO f MCre) reduced tumor burden (g) compared to control MCre mice on Days 9 of tumor implant (n = 14 mice per group). **(D)** Representative figure of FACS analyses of splenic tissue confirmed reduction of Breg (CD19^+^CD5^+^CD1d^hi^IL-10^+^) in tumor-bearing IDO f Mcre on day 9 post tumor implant. **(E)** Diminished Breg frequency in IDO f Mcre mice with lung cancer compared to MCre control on Days 9 post tumor implant (n = 8 mice per group). Total percentage of Bregs (CD19^+^CD5^+^CD1d^hi^IL-10^+^) were determined by flow cytometry analysis. **(F)** Representative FACS plot of tumor tissue confirming reduction of Breg in IDO f MCre on day 9 of tumor implant. **(G)** Reduced Breg frequency in lung tumor of IDO f MCre mice compared to MCre control on day 9 of tumor implant (n = 10-11 mice per group). *p < 0.05, **p < 0.001, ***p < 0.0001.

The immunosuppressive properties of Bregs have been attributed to IL-10 (18, 50, 51). We quantitated serum levels of IL-10, which was elevated in tumor-bearing WT compared to IDO deficient mice **(** **Supplementary Figure 1B** **)**. We then examined a potential direct role for MDSC-associated IDO as a driver of Breg differentiation in co-cultures of immune sorted MDSCs (CD11b^+^Gr-1^+^) from splenocytes and tumors of tumor implanted WT and IDO^-/-^ mice with CD19^+^ purified B cells in 5:1 ratio (5 B cells: MDSC) **(** **Figures 2A, B** **)**. Impairment of Breg differentiation was observed in B cells co-cultured with MDSCs from IDO^-/-^ compared to WT mice **(** **Figures 2A, B** **)**. Breg differentiation was significantly reduced when MDSCs (CD11b^+^Gr-1^+^) sorted from splenocytes of tumor-bearing IDO^-/-^ mice were co-cultured with total splenocytes (5:1 ratio) compared to WT **(** **Supplementary Figures 1C, D** **)**. Further, we validated the contribution of MDSC-associated IDO in Breg differentiation using IDO f MCre mice with conditional deletion of IDO in myeloid-lineage cells **(** **Supplementary Figure 1E** **)** and their MCre control mice. At day-9 post tumor implant, IDO f MCre mice exhibited significantly lowered tumor burden compared to controls **(** **Figure 2C** **)**. Importantly, we observed a significant reduction in Breg in tumor-bearing IDO f MCre compared to their MCre control **(** **Supplementary Figure 2A** and **Figures 2D–G** **)**. Collectively, these data suggest that Trp catabolizing enzyme IDO plays a major role in Breg differentiation in the lung TME with a significant contribution from MDSC-associated IDO.

### Trp-Metabolite L-Kyn Promotes Breg Differentiation Through AhR Pathway

**Figure 3:**
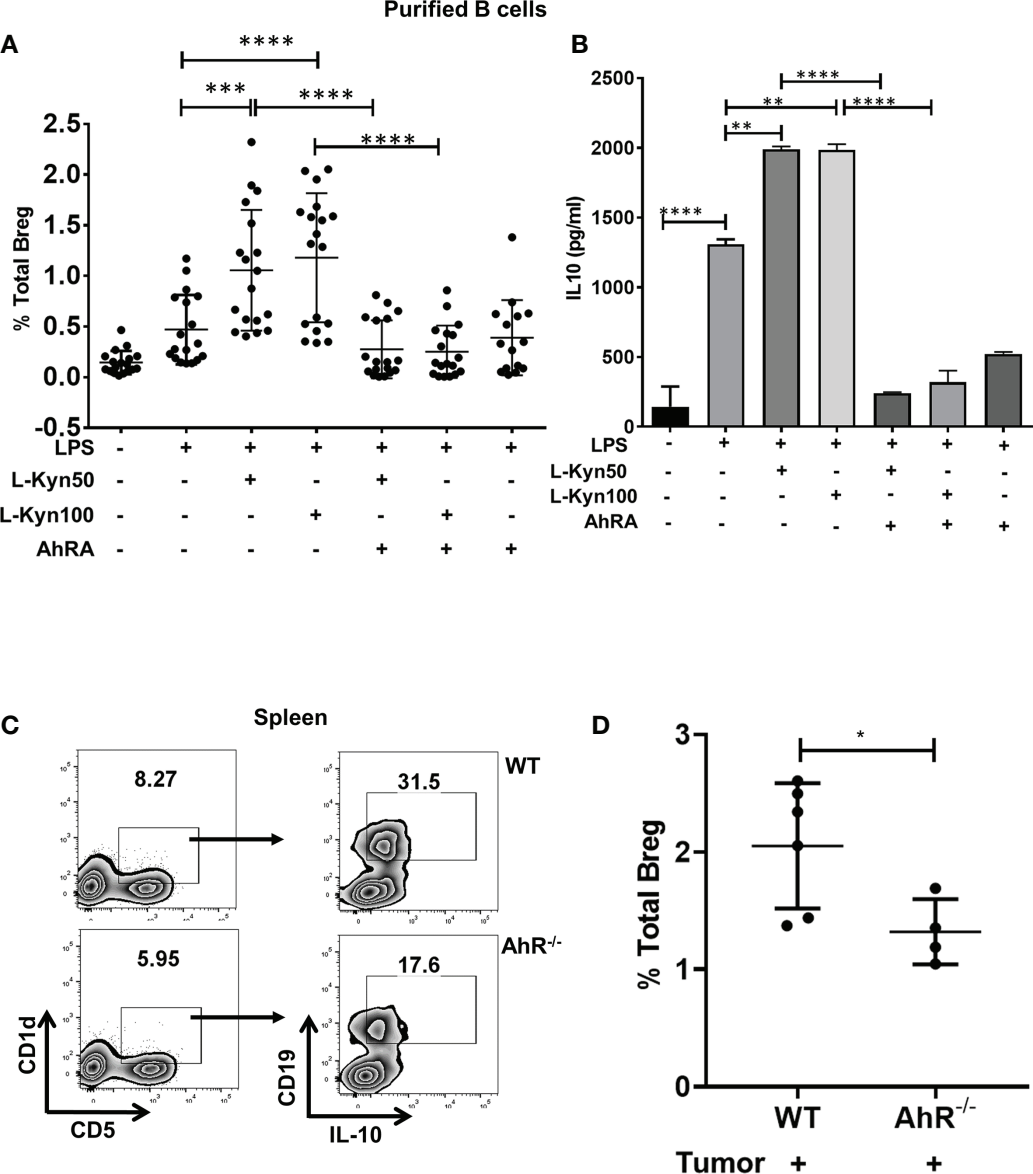
Trp metabolite L-Kyn promoted Breg differentiation through AhR pathway. CD19^+^ B cells were negatively selected from whole splenocytes of WT mice, disaggregated and seeded with LPS (10μg/ml), increasing concentrations of L-Kyn (50µM and 100μM) and LPS + L-Kyn + CH 223191(10μM) an aryl hydrocarbon receptor antagonist (AhRA) for 72 hrs. Bregs were identified as CD19^+^CD5^+^CD1d^hi^IL-10^+^ by flow cytometry. **(A)** Total percentage of Bregs (CD19^+^CD5^+^CD1d^hi^IL-10^+^) in *ex-vivo* experiments performed with negatively selected purified B cells following stimulation with no LPS, LPS, LPS+L-Kyn, LPS+AhRA+L-Kyn, and LPS+AhRA. Data shown here are pooled from six independent experiments with 3 technical replicates in each condition. **(B)** IL-10 levels in the media collected from above described experiments. **(C)** Representative figure of FACS analyses of splenic tissue confirmed the reduction of Breg (CD19^+^CD5^+^CD1d^hi^IL-10^+^) in tumor-bearing AhR^-/-^ on day 9 post tumor implant. **(D)** Total percentage of Bregs (CD19^+^CD5^+^CD1d^hi^IL-10^+^) in spleens of tumor-bearing WT and AhR^-/-^ mice on Day 9 post tumor implant (n = 4-6 mice per group). *p < 0.05, **p < 0.001, ***p < 0.0001, ****p < 0.00001.

IDO pathway metabolite L-Kyn has been reported to be immunosuppressive by promoting Treg through AhR activation (45, 52). Since IDO deficiency resulted in impaired Bregs frequency in lung TME, we evaluated whether L-Kyn promotes Breg differentiation through AhR. In *ex-vivo* cultures of CD19^+^ B cells purified from splenocytes of naïve WT mice stimulated with no LPS controls and LPS+Kyn and LPS+Kyn+AhRA (AhR antagonist, CH-223191, AhRA) treatments, we observed induction of CD19^+^CD5^+^CD1d^hi^IL-10^+^ Bregs frequency in response to stimulation with LPS+L-Kyn while Breg differentiation was inhibited with the addition of AhRA **(** **Supplementary Figures 3A, B** and **Figure 3A** **)**. In addition, we detected elevated levels of IL-10 in the culture supernatant from LPS+Kyn treated samples (**Figure 3B** **)**. These data suggest that L-Kyn promotes Breg through AhR pathway. Furthermore, CD19^+^CD5^+^CD1d^hi^ Breg precursors were elevated following treatment with LPS+L-Kyn in a dose-dependent manner. But the expansion of these Breg precursors was present even in the presence of AhRA compared to LPS only stimulation *ex-vivo* **(** **Supplementary Figure 4A** **)**, demonstrating that L-Kyn induced Breg precursor differentiation was AhR independent. We then evaluated the role of L-Kyn alone without LPS, and found that Breg precursors (CD19^+^CD5^+^CD1d^hi^) were enhanced, but L-Kyn alone was unable to transform them into IL-10 producing Breg, suggesting that LPS stimulation was essential to induce Breg precursors to become IL-10 producing Breg *ex-vivo* **(** **Supplementary Figures 4B, C** **).** Our qRT-PCR data validated this finding **(** **Supplementary Figure 3C**). Furthermore, we observed enhanced Breg frequency also in *ex-vivo* cultures of whole splenocytes stimulated with LPS+L-Kyn compared to only LPS controls **(** **Supplementary Figure 4D** **)**. We confirmed that the viability of CD19^+^ purified B cells was not affected following stimulation with either L-Kyn over a range of 50 to 500µM concentration with or without LPS and AhRA **(** **Supplementary Figure 5** **)**. Thus taken together, inhibition in Breg frequency was due to the inactivation of AhR rather than the toxicity of L-Kyn or AhRA. Further, we validated the contribution of AhR in Breg differentiation using AhR^-/-^ mice at day-9 post tumor implant, AhR^-/-^ mice exhibited significantly lowered Breg frequency compared to WT control mice **(** **Supplementary Figure 2B** and **Figures 3C, D** **)**.

**Figure 4:**
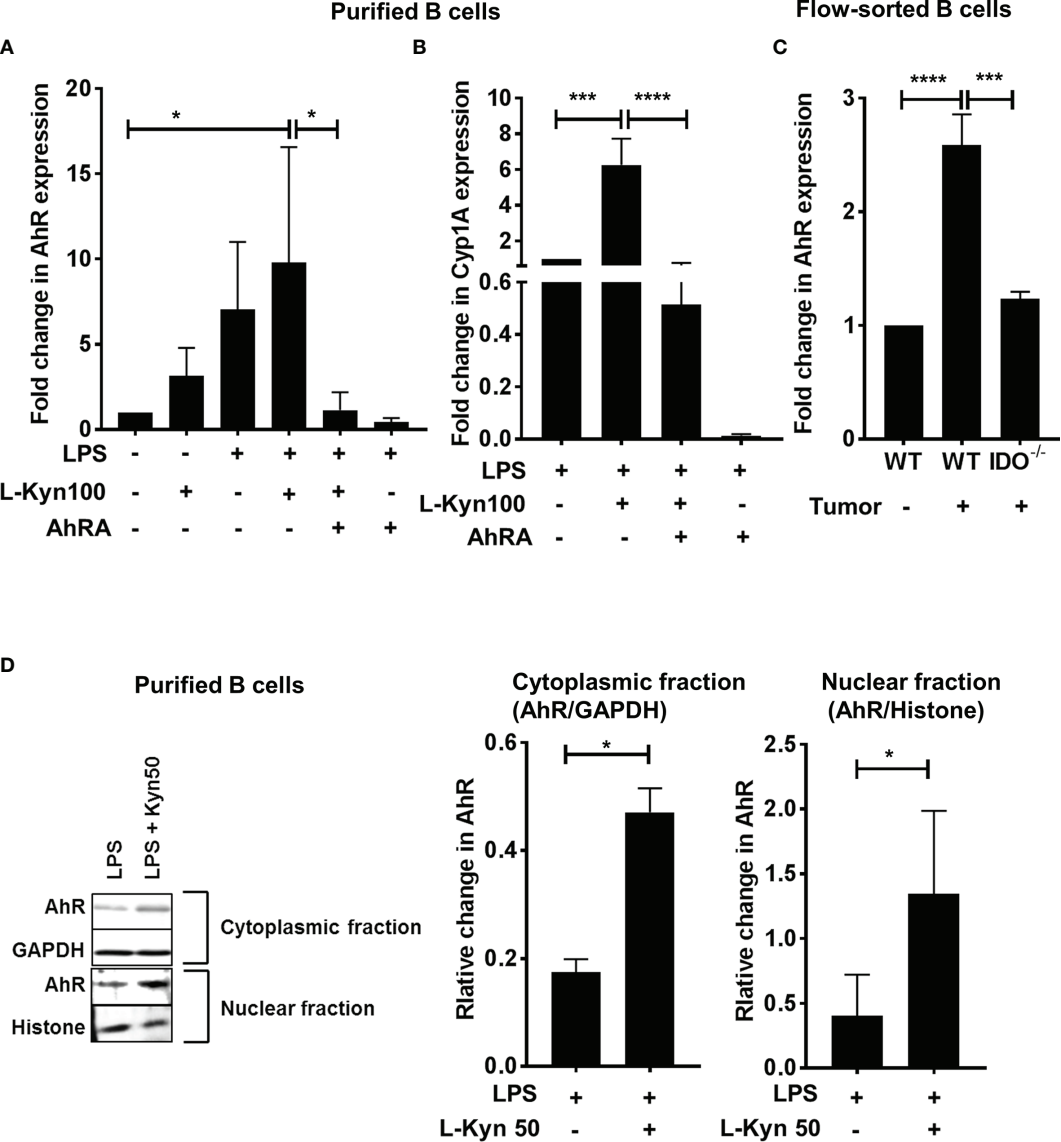
Trp metabolite L-Kyn activated AhR pathway. CD19^+^ B cells were negatively selected from whole splenocytes of WT mice, disaggregated and seeded with LPS (10μg/ml), L-Kyn (50µM and 100μM) and LPS + L-Kyn + CH 223191(10μM) an aryl hydrocarbon receptor antagonist (AhRA) for 72 hrs **(A)** AhR expression was assessed by qRT-PCR in the cells collected from *ex-vivo* experiments performed with purified B cells in experimental conditions described above **(B)** AhR target gene Cyp1A1 expression was assessed by qRT-PCR in purified B cells *ex-vivo* experiments following stimulation of LPS, LPS+L-Kyn, LPS+L-Kyn+AhRA and LPS+AhRA **(C)** AhR expression was assessed by qRT-PCR in B cells (CD19^+^) sorted from naïve WT, tumor-bearing WT and tumor-bearing IDO^-/-^ mice on Day 9 post tumor implant **(D)** Cytoplasmic and nucleoplasmic fraction were collected from purified B cells from *ex-vivo* experiments following stimulation with LPS and LPS+L-Kyn Western blot analysis was performed to detect AhR expression in cytoplasmic and nucleoplasmic fractions and the relative expression was normalized with GAPDH and histone respectively. *p < 0.05, ***p < 0.0001, ****p < 0.00001.

To further ascertain the role of AhR, we determined the expression of AhR and its target gene Cyp1A1 by qRT-PCR in purified CD19^+^ B cells stimulated with either LPS, LPS+L-Kyn or LPS+L-Kyn+AhRA. Both AhR and Cyp1A1 gene expression was upregulated in B cells stimulated with LPS+L-Kyn **(** **Figures 4A, B** **)** compared to controls. Additionally, we confirmed the role of AhR in Breg differentiation in lung cancer, by validating the upregulation of AhR expression in immune sorted CD19^+^ B cells from splenocytes of tumor-bearing WT compared to tumor-bearing IDO^-/-^ and WT naïve **(** **Figure 4C** **).** L-Kyn is an endogenous ligand of cytoplasmic transcription factor AhR, which after binding with AhR makes AhR complex, translocates into the nucleus to bind further with AhR nuclear translocator (Arnt). TheAhR-Arnt heterodimers then finally bind with dioxin-responsive elements (DREs) to regulate gene expression in a variety of cells (47). To obtain mechanistic understanding behind L-Kyn induced Breg differentiation, we assessed if stimulation of B cells with L-Kyn resulted in translocation of AhR to the nucleus. Immunoblot analyses performed with cytoplasmic and nucleoplasmic fractions obtained from LPS and LPS+ L-Kyn stimulated B cells showed induction of AhR expression **(** **Figure 4D**), suggesting a critical role of AhR in L-Kyn induced Breg differentiation. We then investigated if AhR deficiency in B cells impairs L-Kyn induced Breg differentiation. Purified CD19^+^ B cells from splenocytes of naïve AhR^-/-^ were cultured to perform *ex-vivo* experiments followed by LPS+L-Kyn stimulation (53).

**Figure 5:**
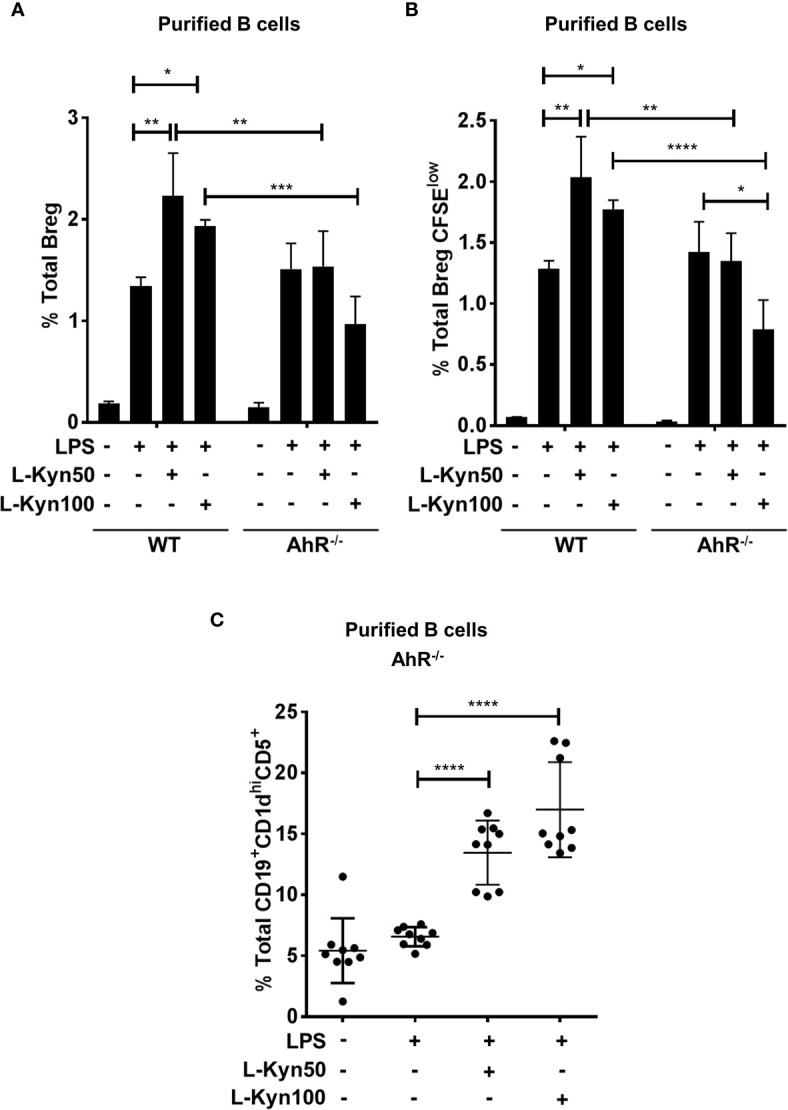
AhR deficiency in B cells impaired Breg differentiation *ex-vivo*. CD19^+^ B cells were negatively selected from whole splenocytes of wild-type (WT), and AhR^-/-^, mice were disaggregated and seeded with LPS (10μg/ml), and increasing concentrations of L-Kyn (50µM and 100μM) for 72 hrs. Bregs were identified as CD19^+^CD5^+^CD1d^hi^IL-10^+^ by flow cytometry. **(A)** AhR deficiency inhibited L-Kyn induced Breg differentiation following stimulation of B cells with LPS+L-Kyn. Data shown here are representative of three independent experiments with three replicates for each group and represent the mean ± STDEV values. **(B)** AhR deficiency altered the proliferation of Breg. CD19^+^ cells were negatively selected from whole splenocytes of WT, and AhR^-/-^ mice and labeled with CFSE and then stimulated with LPS and increasing concentrations of L-Kyn for 72 hrs. Total percentages of Breg^+^CFSE^low^ (CD19^+^CD5^+^CD1d^hi^IL-10^+^CFSE^low^) cells were determined by flow cytometry analysis. Data shown here are representative of two independent experiments with 3 technical replicates in each condition. **(C)** Breg precursors (CD19^+^CD1d^hi^CD5^+^) induced by LPS+L-Kyn were not impaired in AhR^-/-^. Total percentages of CD19^+^CD1d^high^CD5 in AhR^-/-^ were determined by flow cytometry analysis. Data shown here are pooled of three independent experiments with 3 technical replicates in each condition. *p < 0.05, **p < 0.001, ***p < 0.0001, ****p < 0.00001.

Interestingly LPS+ L-Kyn was unable to induce Breg differentiation in AhR^-/-^ B cells, and in addition, a significant reduction in Bregs frequency was also observed in AhR^-/-^ compared to WT control mice **(** **Figure 5A** **)**. Furthermore, we found diminished *ex-vivo* proliferation in CFSE stained Bregs of AhR^-/-^ compared to their WT controls followed by LPS and LPS+L-Kyn stimulation, as described above **(** **Figure 5B** **)**. Additionally, consistent with our previous finding **(** **Supplementary Figure 4A** **)**, we observed induction in frequency of Breg precursors (CD19^+^CD5^+^CD1d^hi^) in AhR deficient CD19^+^ B cells upon stimulation with LPS+L-Kyn compared to LPS only stimulation (**Figure 5C** **)**, strongly supporting our hypothesis that L-Kyn induced Breg precursor differentiation is AhR independent.

CD40,TLRs, or cytokine-mediated activation turns immature B cells directly into Breg or immature B cells may first differentiate into mature B cells and then into IL-10 producing Breg (54). Therefore we investigated if L-Kyn modulated specific B cell subsets to differentiate into Breg. Interestingly, we observed that only mature B cells (CD19^+^IgM^+^IgD^+^) upon stimulation with LPS+L-Kyn induced frequency of CD19^+^CD5^+^CD1d^hi^ Breg precursors **(** **Supplementary Figure 6** **)**. Collectively these findings suggest that L-Kyn, an IDO-derived Trp metabolite activates AhR and translocate AhR complex into the nucleus of B cells to drive their differentiation into IL-10, producing Breg. At the same time, L-Kyn alone enhances the Breg precursor pool *ex-vivo*, which upon stimulation with LPS transform them into IL10 producing Bregs.

### L-Kyn Promotes Breg Differentiation in TLR-4-MyD88 Dependent but TLR-2 Independent Manner While Breg Precursor Differentiation Is Independent of TLR-2 and TLR-4

**Figure 6:**
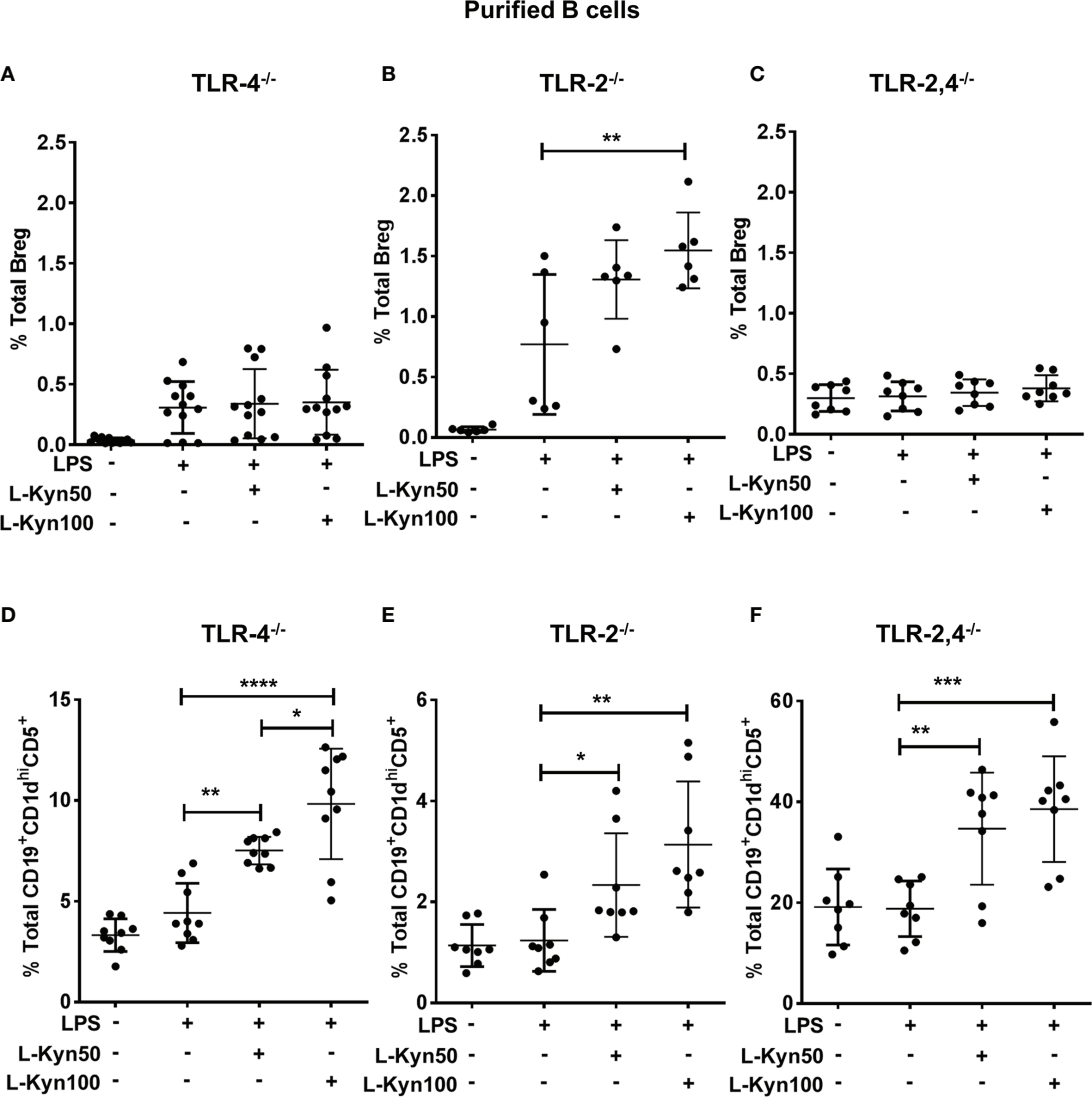
L-Kyn induced Breg differentiation was TLR-4 dependent but TLR-2 independent, the differentiation of Breg precursor was TLR-2 and TLR-4 independent. CD19^+^ B cells were negatively selected from whole splenocytes of TLR-4^-/-^, TLR-2^-/-^ and TLR-2,4^-/-^ mice were disaggregated and seeded with LPS (10μg/ml), and increasing concentrations of L-Kyn (50µM and 100μM) for 72 hrs. Bregs were identified as CD19^+^CD5^+^CD1d^hi^IL-10^+^ by flow cytometry. **(A, C, E)** Upper panel- L-Kyn induced Breg differentiation was impaired in B cells of TLR-4^-/-^ and TLR-2, 4^-/-^ but induction in TLR-2^-/-^ following stimulation with LPS+L-Kyn. Data shown here are pooled from four independent experiments with 3 technical replicates in each condition. **(B, D, F)** Lower panel- L-Kyn promoted Breg precursors (CD19^+^CD1d^hi^CD5^+^) in B cells of TLR-4^-/-^ TLR-2^-/-^ and TLR-2, 4^-/-^ following stimulation with LPS+L-Kyn. Data shown here are pooled from three independent experiments with 3 technical replicates in each condition *p < 0.05, **p < 0.001, ***p < 0.0001, ****p < 0.00001.

It has been already established that LPS dependent activation in immune cells is mainly mediated by TLR-4 through CD14 *via* transcription factor NF- κB to regulate several genes associated with the expression of antigen-presenting molecules, cytokines, transcription factors and enzymes including IDO (55, 56). We next elucidated the potential role of TLRs in L-Kyn induced Breg differentiation. To functionally characterize these using naïve TLR-4 deficient mice, we analyzed Breg as well as Breg precursors in *ex-vivo* co-cultures of CD19^+^ B cells purified from splenocytes of TLR-4^-/-^ mice. We observed no significant difference in Breg but a significant increase in Breg precursors in cultures with TLR-4 deficient cells **(** **Figures 6A, B** **)**, suggesting that TLR-4 signaling may not be necessary for differentiation of B reg precursors. But TLR-4 signaling was important for transforming Breg precursors into IL-10 producing Breg. Additionally, LPS-TLR-4 interaction resulted in MyD88 dependent activation of L-Kyn induced Breg differentiation **(** **Supplementary Figures 7A, B** **),** collectively demonstrating that LPS+L-Kyn induced Breg differentiation is TLR-4-MyD88 dependent, but Breg precursor differentiation is TLR-4 independent.

Previously, LPS induced TLR-2 activation and TLR-2 dependent Breg induction have been reported (55, 57). To determine whether TLR-2 influences L-Kyn induced Breg differentiation *ex-vivo*, we stimulated CD19^+^ purified B cells from TLR-2^-/-^ mice with LPS+L-Kyn. Both Breg as well as Breg precursors increased in response to LPS+L-Kyn compared to only LPS stimulation **(** **Figures 6C, D** **).** These data suggested that L-Kyn induced Breg precursors as well as Breg differentiation, may be TLR-2 independent. Important**ly** this was confirmed with CD19^+^ purified B cells from TLR-2,4 double knock out (TLR-2,4^-/-^) mice (**Figures 6E, F**). It is unclear if in the absence of TLR-2, compensatory signaling may enhance B reg precursor and B reg differentiation. The total percentage of Breg (CD19^+^CD5^+^CD1d^hi^IL-10^+^) was analyzed in *ex-vivo* experiments performed with negatively selected purified B cells from WT, MYD88^-/-^ and TLR2^-/-^ following stimulation with high concentration of L-Kyn (100μM) alone compared to LPS alone. No significant change in Breg was noted among WT, MYD88^-/-^ and TLR2^-/-^ in absence of LPS (**Supplementary Figure 7B** **)**. While the Breg precursors at baseline were significantly higher in the TLR-2,4^-/-^ deficient mice compared to the individual KO, this did not correlate with baseline B reg numbers **(** **Figure 6F** **)**.

### IDO Negatively Regulates B Cell Proliferation and Function

We then investigated if the reduction in B reg differentiation is due to alteration of overall B cell differentiation resulting from IDO deficiency. As we had reported earlier that B cell differentiation is impaired during tumor growth in IDO sufficient mice (34), we evaluated this in IDO deficient mice. We determined if IDO deficiency affect B cell development in tumor-bearing mice. Both percentages and absolute numbers of total B220^+^ cells, B220^+^IgD^-^IgM^-^CD24^int^CD43^+^ pro-B cells, B220^+^IgD^-^IgM^-^ CD24^hi^CD43^-^ pre-B and B220^+^IgD^+^IgM^+^ mature B cells were decreased in BM of tumor-bearing IDO deficient mice compared with naïve IDO deficient mice **(** **Supplementary Figures 8A, B, 9A, B** **)**. Furthermore, both the percentages and numbers of B220^+^CD93^+^ immature B cells were increased, whereas B220^+^CD93^-^CD21^int^CD23^+^ follicular B cells were decreased in tumor-bearing IDO deficient mice compared with naïve IDO deficient mice **(** **Supplementary Figures 10A, B** **)**. However, we observed no difference in total B cells, pro-B cells, pre-B cells, immature B cells and mature B cells in BM **(** **Supplementary Figures 9A, B** **)**, as well as total B cells, immature B cells, marginal zone B cells and follicular B cells in spleens **(** **Supplementary Figures 10A, B** **)** between WT and IDO naïve mice or tumor-bearing mice. These results indicate that IDO deficiency does not affect B cells development in BM and spleen both in naïve tumor-free mice and tumor-bearing mice.

**Figure 7:**
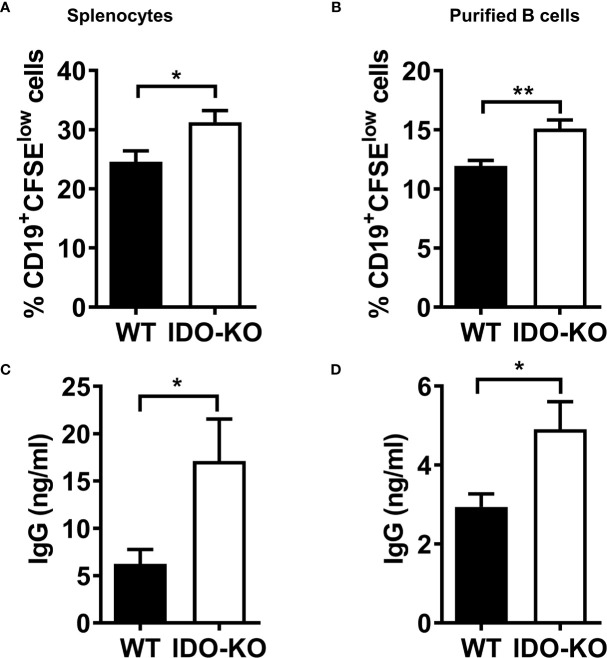
IDO negatively regulate B cell proliferation an IgG production. **(A, B)** Splenocytes **(A)** or CD19^+^B220^+^ B cells sorted **(B)** from tumor-bearing mice on day 9 post-LLC i.c. challenge were labeled with CFSE and cultured with LPS (20 µg/ml) and IL-4 (10 ng/ml). The percentages of CD19^+^CFSE^low^ cells were determined by FACS analysis on day 4 of culture. **(C, D)** culture **(C)** or purified B cells **(D)** on day 4 of culture. The levels of IgG in the culture supernatants were determined by ELISA. *P < 0.05, **P < 0.01.

It has been reported that B cell-intrinsic IDO regulates humoral immunity to T cell-independent antigens (58). To investigate whether IDO regulates B cell proliferation *in vitro*, splenocytes or sorted CD19^+^B220^+^ B cells from WT or IDO^-/-^ tumor-bearing mice were labelled with CFSE and cultured in the presence of LPS and IL-4. We observed that the percentages of CD19^+^CFSE^low^ cells were increased in splenocytes **(** **Supplementary Figure 11** and **Figure 7A** **)** or purified B cells **(** **Figure 7B** **)** from IDO mice compared with that from WT mice. IgG production in culture supernatants was elevated in activated splenocytes **(** **Figure 7C** **)** and/or purified B cells **(** **Figure 7D** **)** from IDO^-/-^ mice compared with WT controls. These results suggest that IDO negatively regulates B cell proliferation and IgG production in tumor-bearing mice.

## Discussion

In this study, we demonstrate that splenic Breg frequency increases with the progression of lung tumor growth in a syngeneic model of lung cancer. Additionally, we report for the first time that IDO associated with tumors and MDSCs promote Breg differentiation during lung cancer progression. Our investigations suggest that IDO-induced Trp metabolite L-Kyn mediates the development of Breg precursors that differentiate into IL-10 producing Breg upon stimulation with LPS *ex-vivo*. Further, we report an uncharacterized role for AhR in regulating L-Kyn induced Breg differentiation. Our *ex-vivo* data generated from TLR-2, TLR-4, TLR-2, 4, and MyD88 deficient B cells indicate that L-Kyn induces Breg differentiation through TLR-4-MyD88 pathway *ex-vivo* while L-Kyn promotes Breg precursors independent of TLR-2 and TLR-4 signaling. Furthermore, our *ex-vivo* B and T cells co-culture studies support our conclusion that L-Kyn induces Breg differentiation independent of T cells. Importantly, we provide evidence from *ex-vivo* experiments that tumor antigens modulated by IFN-γ induces IDO, which then promotes antigen-specific Breg differentiation through AhR pathway. Collectively, these data conclude that IDO, a Trp metabolizing enzyme, and L-Kyn, a major metabolite of Trp promote Breg differentiation through AhR and TLR-4-MyD88 pathways.

Immune cell expression of IDO is dominated by immunosuppressive cell types including T_regs_, MDSCs, and pDCs and very recently reported also by macrophages (59–62). IDO^+^ dendritic cells can impair antigen presentation from antigen-presenting cells (APCs) in the tumor-draining lymph nodes to recruited effector T cells. Furthermore, tumor cells that express IDO not only promote Treg differentiation but also disable effector T cell function (45). The concept that Breg orchestrate immune modulation was first suggested 4 decades ago, when B cell depleted splenocytes were associated with the increased hypersensitivity, suggesting a positive correlative loop between Breg and Treg (63, 64).

The mechanisms underlying the augmentation of Breg infiltration with lung cancer progression are largely unknown. Previously, researchers have reported corroborative evidence regarding correlation of Breg infiltration with cancer progression (65, 66). Breg have been implicated in the clinical progression of various types of cancers and have been proposed to promote tumor growth through IL-10 production (67–69).To date, relatively little is known about Breg differentiation in the lung TME. Previous studies have shown that chemoattractant may attract naïve B cells into TME and promote their differentiation into Breg through a series of soluble mediators (IL-1β, IL-6, IL-21 and IL-35) and cell interaction dependent mechanism (CD40-CD40-L) (54, 70, 71). The induction of Breg was not completely suppressed upon blockade of CD40 (71), thereby indicating that some other factors may likely be involved in Breg differentiation.

We and others have reported previously that in addition to tumor cells, immunosuppressive MDSCs are the major contributors of IDO, a rate-limiting enzyme of Trp catabolism in the TME (41, 42, 72, 73). The impact of MDSC- associated IDO on T cells is well known, but its effect on several subtypes of B cells remains to be determined. It has been hypothesized that MDSCs and tumor cell-associated IDO impairs effector T cell function by promoting differentiation of Treg that support tumor progression (43, 44). Moreover, recent evidence shows that Breg promotes tumor growth by recruiting Treg that inhibit the infiltration of CD8^+^ T cells and CD49^+^ NK cells within the TME (15). There is increasing evidence that MDSCs regulate B cells indirectly by expansion of Treg through Breg (21, 48, 49, 74–76). We recently reported that MDSCs impair B cells proliferation and function during lung tumor progression while differentiation of immunosuppressive Breg was increased (34). It is not known whether the MDSC supported milieu suppresses normal B cell differentiation, instead drives Breg differentiation. Park et al. assessed the impact of MDSCs in Breg differentiation in the murine model of systemic lupus erythematosus (SLE). They proposed that MDSCs induce Breg differentiation *via* iNOS and ameliorate autoimmunity (77). Interestingly, we observed that MDSCs suppress B cell responses *via* iNOS, and it is possible that the iNOS induction while suppressing B cell proliferation promotes Breg (34). To the best of our knowledge, MDSCs-mediated regulation of the function of Breg has not been studied in the context of cancer. In addition, none of these prior studies have identified that IDO produced from MDSCs and tumor cells is involved in Breg expansion. Our co-culture data obtained from IDO deficient MDSCs and B cells demonstrate for the first time that IDO associated MDSCs induces Breg *ex-vivo.* In addition, reduced Breg frequency in a syngeneic tumor model with conditional deletion of IDO in myeloid cells (IDO f MCre) further support the positive regulation by MDSC- associated IDO in Breg differentiation *in-vivo*. Therefore our current studies and previous evidence are in resonance with our observations that IDO from MDSCs and tumor cells promote Breg during lung cancer progression.

Induction of IDO activity in the TME results in the depletion of Trp, an essential amino acid needed for T cell proliferation, thus regulating T cell responses (45, 78). IDO-dependent Trp metabolite L-Kyn is a well-known endogenous ligand of cytoplasmic transcription factor AhR (47). Ligand mediated activation of AhR performs a crucial role in regulating gene expression in a variety of cells (47). AhR has long been established as an environmental receptor for dioxins. Additionally, it has been previously reported that L-Kyn activates AhR to induce selective expansion of Treg by activation of forkhead box p3 (Foxp3) in naïve T-cells and also biasing macrophages and dendritic cells (DCs) towards an immunosuppressive phenotype (46, 52, 79, 80). Importantly, AhR activation positively regulates IDO induction in DCs and consequently promotes L-Kyn production (81, 82). L-Kyn mediated AhR activation promotes tumor growth with elevated levels of inflammatory cytokines (IL-6, IL-8, and IL-1β) and a decreased frequency of infiltrating cytotoxic CD8^+^ T cells in TME (47). AhR is also highly induced in subsets of B cells and leads to suppression of humoral immune response upon activation with environmental pollutants (83, 84). There is emerging evidence that AhR plays a critical role in activation-induced cell fates resulting in class switching of B cells (85). Recently, Matteo et al. have shown that mature B cells express AhR upon ligand-specific activation by AhR translocation to the nucleus resulting in induction of downstream target gene Cyp1A1 (86). Some of tumor-derived metabolites such as 5-lipoxygenase in a breast cancer model and placental growth factor (PIGF) in gliomas have been proposed to promote Breg (87, 88). To date, prior studies have not identified a role for L-Kyn mediated AhR activation in Breg differentiation. Consistent with these prior studies, we put forth new evidence that L-Kyn activates AhR in mature B cell subsets by translocating AhR into their nucleus and helping them differentiate into Breg precursors, and furthermore, TLR-4 signaling transforms them into IL-10 producing Breg.

Immune cell activation by LPS is mainly mediated by TLR-4 (55). TLR ligands, such as LPS and CpG are potent AhR agonists in various cell types (82). Previously TLRs signaling was thought to be directly involved in modulating the regulatory function of B cells (32). Evidences suggest that B cells produce IL-10 upon stimulation with LPS (51, 89). It has been previously reported that TLR-4, TLR-2, and adaptor MyD88 are directly involved in modulating the regulatory function of B cells in a murine model of experimental autoimmune encephalomyelitis (EAE) (32). Our *ex-vivo* data obtained from TLR-4, TLR-2 and MyD88 deficient B cells are consistent with these prior studies and strongly support our current hypothesis that L-Kyn induces Breg precursors (CD19^+^CD1d^hi^CD5^+^) independent of TLR-2 and TLR-4 signaling but depends on TLR-4-MyD88 pathway to transform them into IL-10 producing Breg cells. Furthermore, our hypothesis is also strongly proven by another *ex-vivo* data showing LPS (TLR-4 ligand) is required to transform Breg precursors into Breg cells.

We reported earlier that Breg cells were increased in the spleens and lungs of wild type tumor-bearing mice (34). The reduction in percentages of Breg in in the spleen and tumor tissue of IDO^-/-^ mice, that we report here, is not due to overall reduction B cell development or differentiation. Although we and others (58) show that IDO deficiency does not affect B cell development in BM and spleen, IDO negatively regulates B cell proliferation and IgG production in tumor-bearing mice. The functional enhancement of B cells with IDO deficiency is also consistent with the reduction in tumor burden in IDO^-/-^ mice (41).

Thus our current study provides valuable insights into mechanisms underlying Breg differentiation, specifically, regulation by IDO (from MDSCs and tumor cells) and by Trp metabolite L-Kyn *via* AhR pathway and highlight a potential link between IDO and Breg development in TME. Enhanced tumor protection was achieved in B cells deficient mice, and the anti-tumor immune response was reduced significantly when B cells were adoptively transferred in those murine models of tumor (15, 90). Considering the immunosuppressive effect of B cells, enhanced efficacy of melanoma vaccine has been observed in the absence of B cells (91). Consistent with these prior studies, our recent most data on Breg raises the new possibility of immunotherapeutic intervention for lung cancer.

## Data Availability Statement

The original contributions presented in the study are included in the article/**Supplementary Material**, further inquiries can be directed to the corresponding author.

## Ethics Statement

The animal study was reviewed and approved by the Institutional Animal Care and Use Committee the University of Alabama at Birmingham, AL, USA.

## Author Contributions

ST, YW, JJ, KH, and JD designed the experiments. ST, YW, JJ, KH, and JS and performed the experiments. ST, YW, and JD prepared figures and supplementary material sections. ST, YW, and JD wrote the manuscript. ST, YW JJ, KH, JS, MA, VT, RM, SP, and JD reviewed and revised the manuscript. All the authors contributed to final data analysis, discussions, and manuscript preparation.

## Funding

This study was supported by institutional support through ACS– IRG-60-001-53-IRG.

## Conflict of Interest

The authors declare that the research was conducted in the absence of any commercial or financial relationships that could be construed as a potential conflict of interest.

## Publisher’s Note

All claims expressed in this article are solely those of the authors and do not necessarily represent those of their affiliated organizations, or those of the publisher, the editors and the reviewers. Any product that may be evaluated in this article, or claim that may be made by its manufacturer, is not guaranteed or endorsed by the publisher.
