# Supplementary material for: Indoleamine 2, 3-Dioxygenase Promotes Aryl Hydrocarbon Receptor-Dependent Differentiation Of Regulatory B Cells in Lung Cancer
Source: Front Immunol. 2021 Nov 19;12:747780. doi: 10.3389/fimmu.2021.747780 (PMC8640488; doi:10.3389/fimmu.2021.747780)
Supplement: Supplementary file 1 [file DataSheet_1.pdf]

Supplementary Figure 1

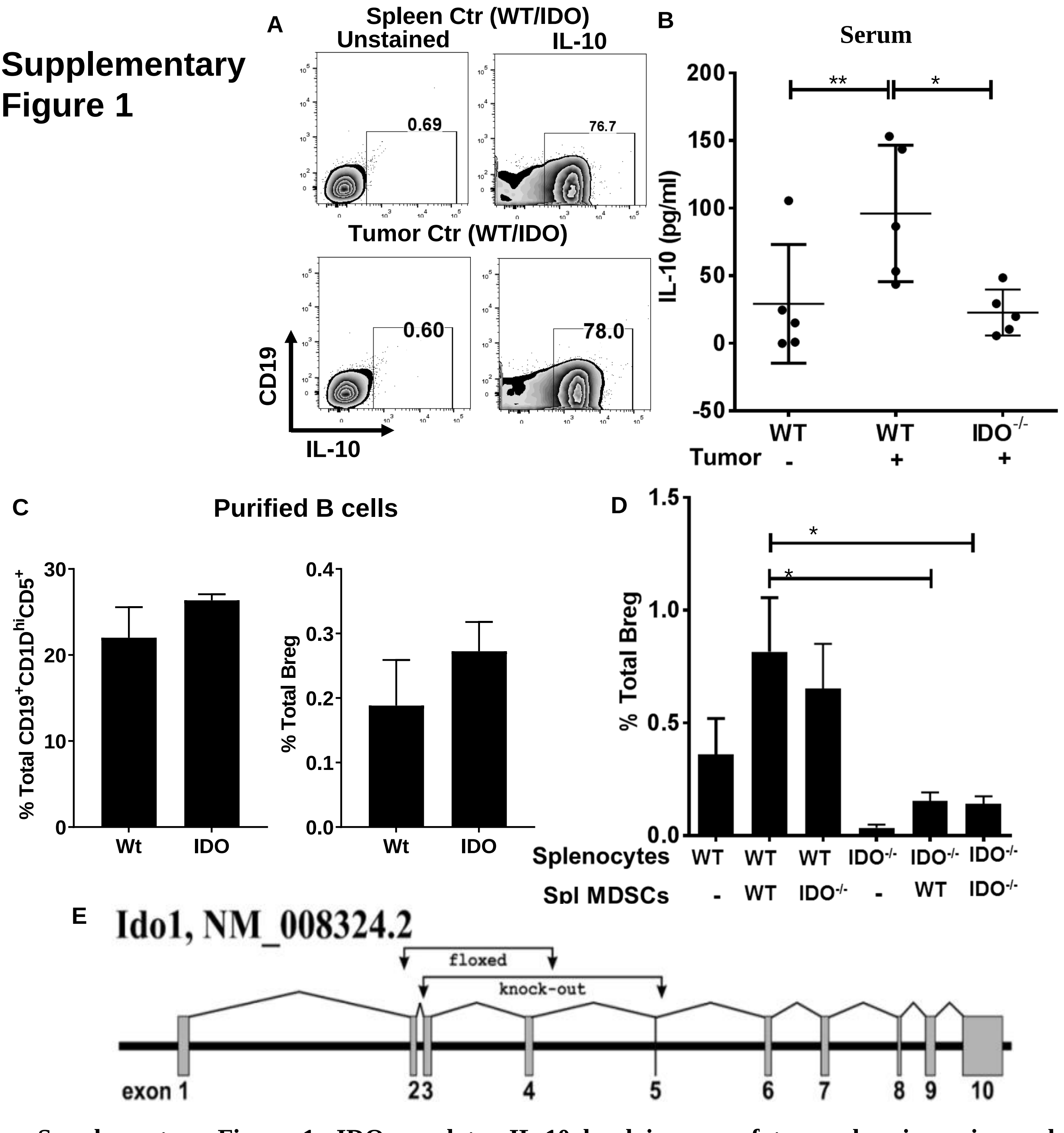

Supplementary Figure 1- IDO regulates IL-10 level in sera of tumor bearing mice and MDSCs mediated IDO promotes Breg differentiation in splenic milieu. (A) Gating strategy of IL-10 in WT/IDO spleen and tumor.**(B)** IL-10 levels were elevated in the mouse sera of tumor bearing WT compared to naïve WT and tumor bearing IDO$^{-/-}$ on day 9 post tumor implant (n=5 mice per group). **(C)** Breg precursors and Breg percentage in purified B cells from WT/IDO$^{-/-}$ before culturing with MDSCs **(D)** Whole splenocytes of WT and IDO$^{-/-}$ naïve mice were co-cultured in 5:1 ratio with MDSCs sorted from spleen of tumor bearing WT and IDO$^{-/-}$ and incubated for 72 hrs. Bregs were identified in the co-culture as CD19^{+}CD5^{+}CD1d^{hi}IL-10^{+} by flow cytometry. Data shown here are representative of two independent experiments with three replicates for each group and represent the mean ± STDEV values. *p < 0.05. **(E)** Figure drawn to approximate scale, illustrates the region targeted for excision by Cre recombinase compared to the region deleted in the JAX Ido1KO strain, 005867 to target Ido1 inactivation in myeloid-derived cells

Supplementary Figure 2

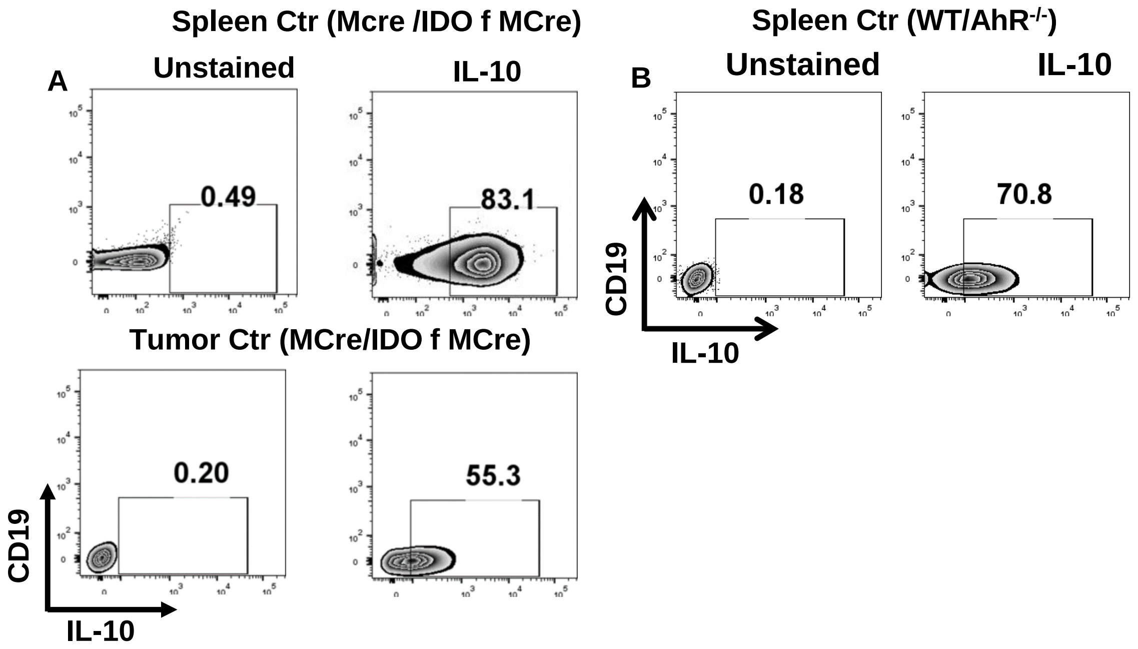

Supplementary Figure 2- (A) Gating strategy of IL-10 in MCre/IDO f MCre spleen and tumor.(**(B)** Gating strategy of IL-10 in WT/AhR$^{-/-}$ spleen

Supplementary Figure 3

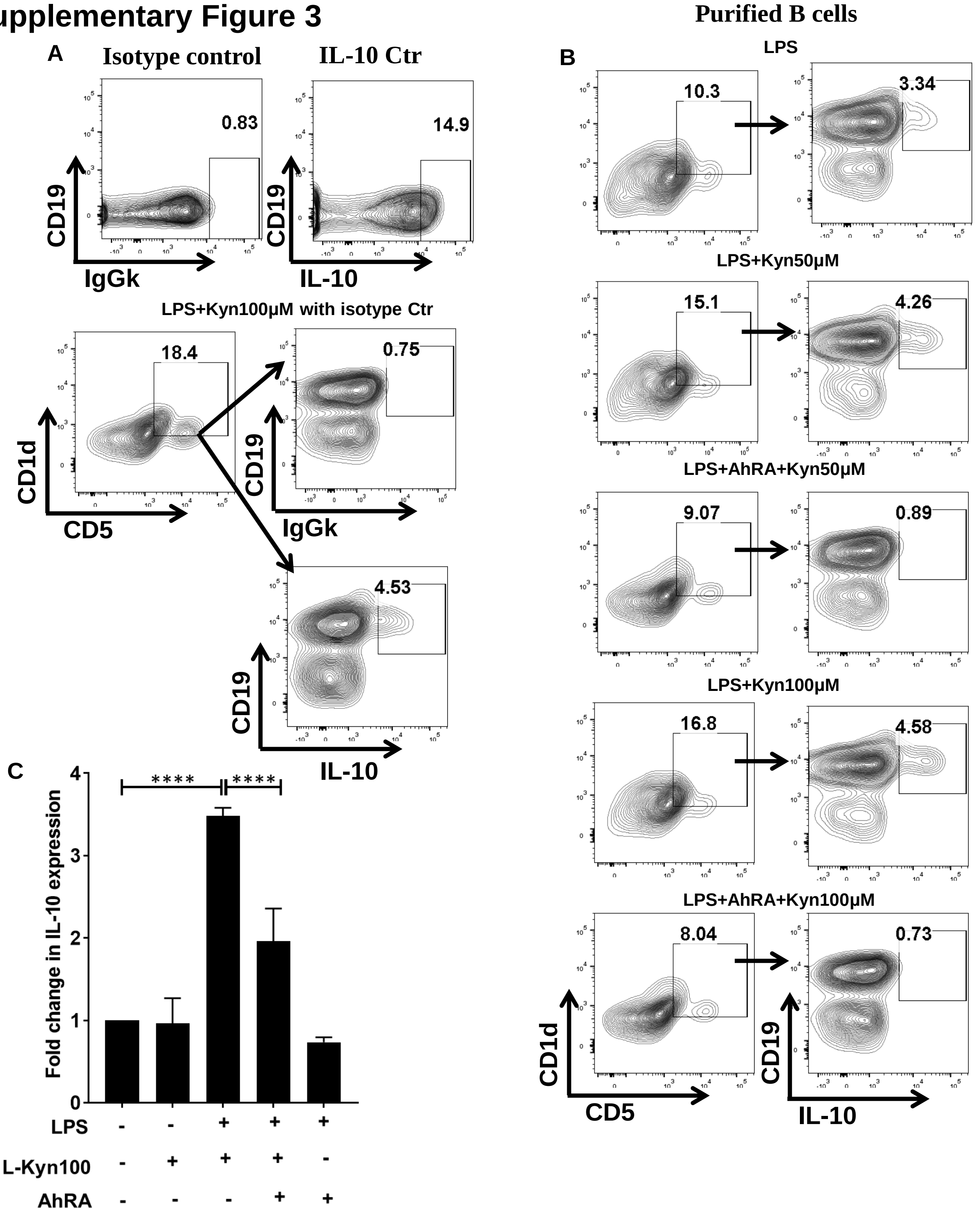

Supplementary Figure 3- L-Kyn promoted IL-10 producing Bregs ($CD19^+CD5^+CD1d^{hi}IL\text{-}10^+$) (A) Representative flow cytometry contour plot showing IL-10 gating with isotype control. **(B)** Representative flow cytometry contour plot showing in-vitro induction of Bregs (CD19+CD5+CD1dhiIL-10+) with LPS+L-Kyn while AhRA with LPS+L-Kyn inhibited Bregs **(C)** $CD19^+$ B cells were negatively selected from whole splenocytes of WT mice, disaggregated and seeded with LPS (10μg/ml), L-Kyn (100μM) and LPS + L-Kyn + CH 223191(10μM) an aryl hydrocarbon receptor antagonist (AhRA) for 72 hrs IL-10 expression was assessed by qRT-PCR in the cells collected from *ex-vivo* experiments performed with purified B cells in experimental conditions described above

Supplementary Figure 4

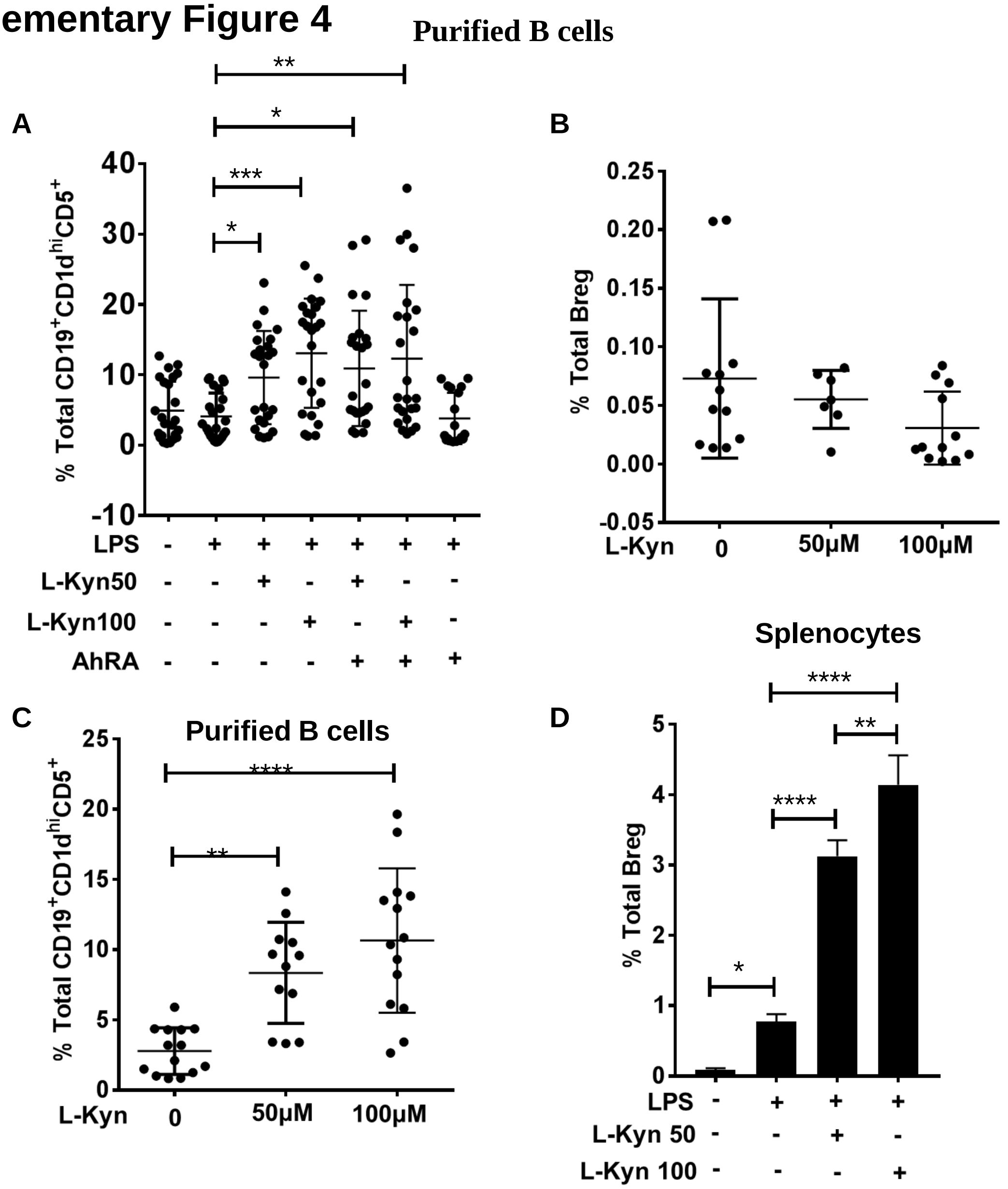

Supplementary Figure 4- L-Kyn promoted Breg precursors (CD19$^+$CD5$^+$CD1d^{hi}) in AhR independent and LPS required to make them Breg. (A) Total percentage of Breg precursors (CD19$^+$CD5$^+$CD1d^{hi}) in *ex-vivo* experiments performed with negatively selected purified B cells following stimulation with no LPS, LPS, LPS+L-Kyn, LPS+AhRA+L-Kyn, and LPS+AhRA. Data shown here are pooled from six independent experiments with 3 technical replicates in each condition **(B)** Total percentage of Breg (CD19$^+$CD5$^+$CD1d^{hi}IL-10$^+$) in *ex-vivo* experiments performed with negatively selected purified B cells following stimulation with no LPS and different concentration of L-Kyn (50µM, 100µM), Data shown here are pooled from 2-4 independent experiments with 3 technical replicates in each condition **(C)** Total percentage of Breg precursors (CD19+CD5+CD1d^{hi}) in ex-vivo experiments performed with negatively selected purified B cells following stimulation with no LPS and different concentration of L-Kyn (50µM, 100µM), Data shown here are pooled from 3-4 independent experiments with 3 technical replicates in each condition. **(D)** Whole splenocytes of WT mice disaggregated and seeded with LPS (10µg/ml) and increasing concentrations of L-Kyn (50µM and 100µM) for 72 hrs. Cells were then stained with antibodies (CD19, CD5, CD1d and IL-10). Flow cytometry was performed to identify Breg. Data shown here are representative of 3 independent experiments with 3 technical replicates in each condition $^*p < 0.05$, $^{**}p < 0.001$, $^{***}p < 0.0001$, $^{****}p < 0.00001$.

Supplementary Figure 5

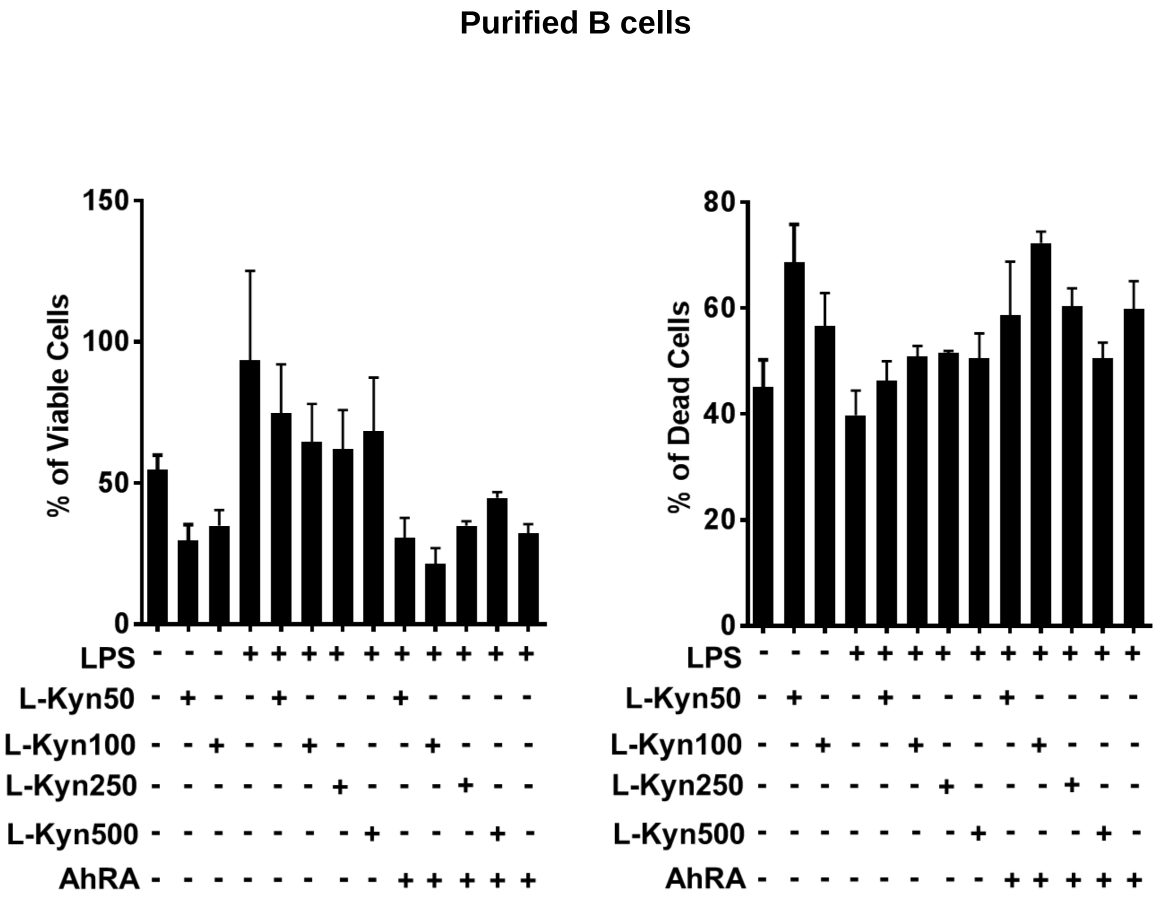

Supplementary Figure 5 – Cytotoxicity of L-Kyn on B cells. $CD19^{+}$ B cells were negatively selected from whole splenocytes of WT mice, disaggregated and seeded with LPS (10µg/ml), increasing concentrations of L-Kyn (50µM, 100µM, 250µM and 500µM) and LPS + L-Kyn + an aryl hydrocarbon receptor antagonist (AhRA) CH 223191 (10µM) for 72 hrs. Viability assay was performed with trypan blue staining to identify live and dead cells.

Supplementary Figure 6

Purified B cells

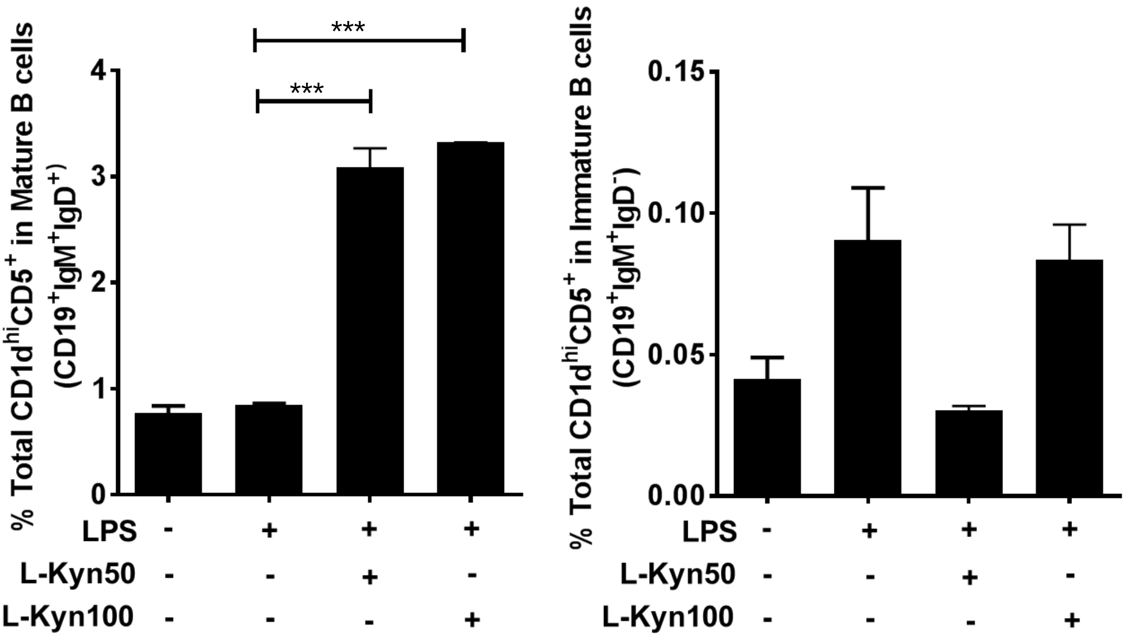

Supplementary Figure 6- L-Kyn differentiated Breg from Mature B cells. Left panel-Total percentage of Breg precursors (CD19+CD5+CD1d^{hi}) from mature B cells (IgM$^+$IgD$^+$) and right panel -Total percentage of Breg precursors (CD19+CD5+CD1d^{hi}) from immature B cells (IgM$^+$IgD$^-$) in ex-vivo experiments performed with negatively selected purified B cells from bone marrow (BM) of WT mice following stimulation with LPS and different concentration of LPS+L-Kyn (50µM, 100µM) *$p < 0.05$, **$p < 0.001$, ***$p < 0.0001$.

Supplementary Figure 7

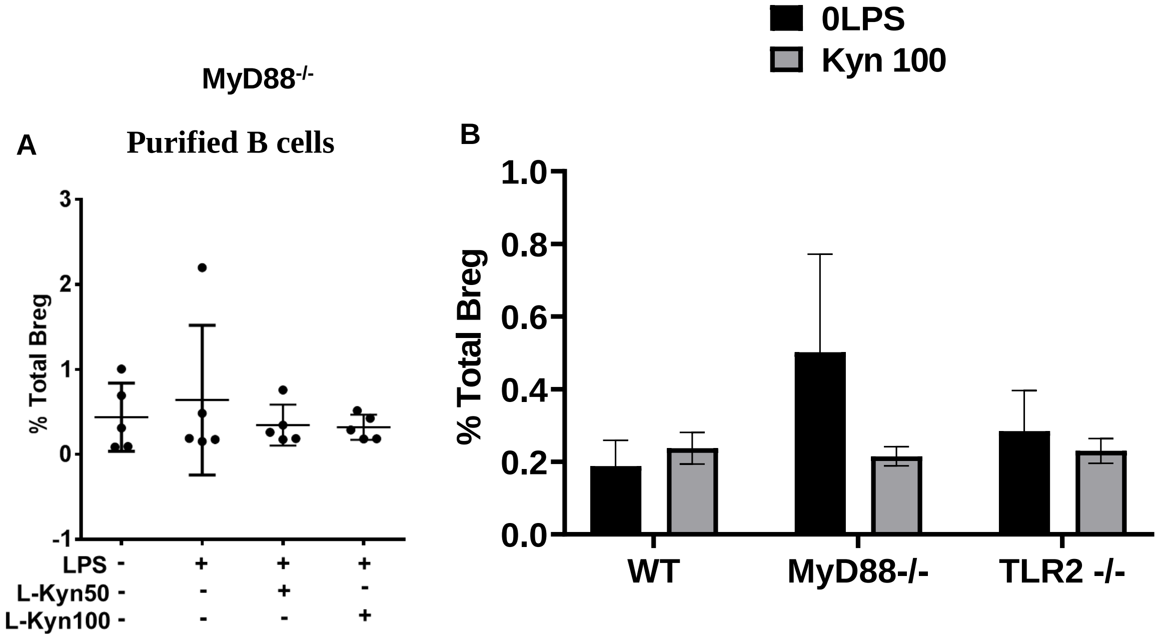

Supplementary Figure 7 - L-Kynurenine induced Breg differentiation was MyD88 dependent (A) L-Kyn induced Breg differentiation was impaired in B cells of MyD88$^{-/-}$ following stimulation with LPS+L-Kyn. (B) No Breg differentiation in B cells of WT, MyD88$^{-/-}$ and TLR2$^{-/-}$ following stimulation with only L-Kyn.

Supplementary Figure 8

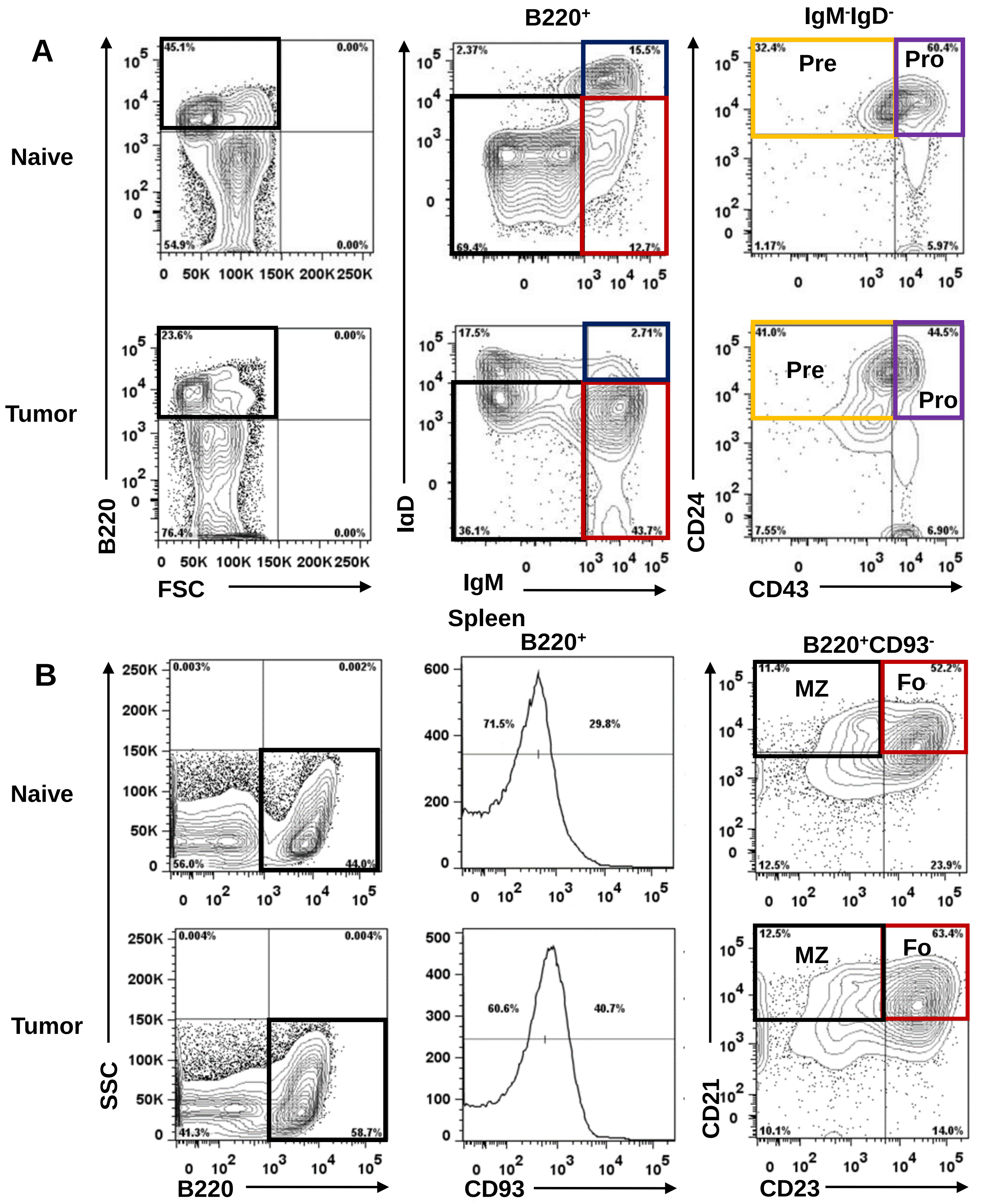

Supplementary Figure 8- Gating strategy of BM and spleen B cell populations. **(A)** Representative gating plots for total B220+ , pro–, pre–, immature, and mature B cells in the BM of naïve and tumor-bearing mice. **(B)** Representative gating plots for total B, immature B, marginal zone B, and follicular B cells in the spleen of naïve and tumor-bearing mice.

Supplementary Figure 9

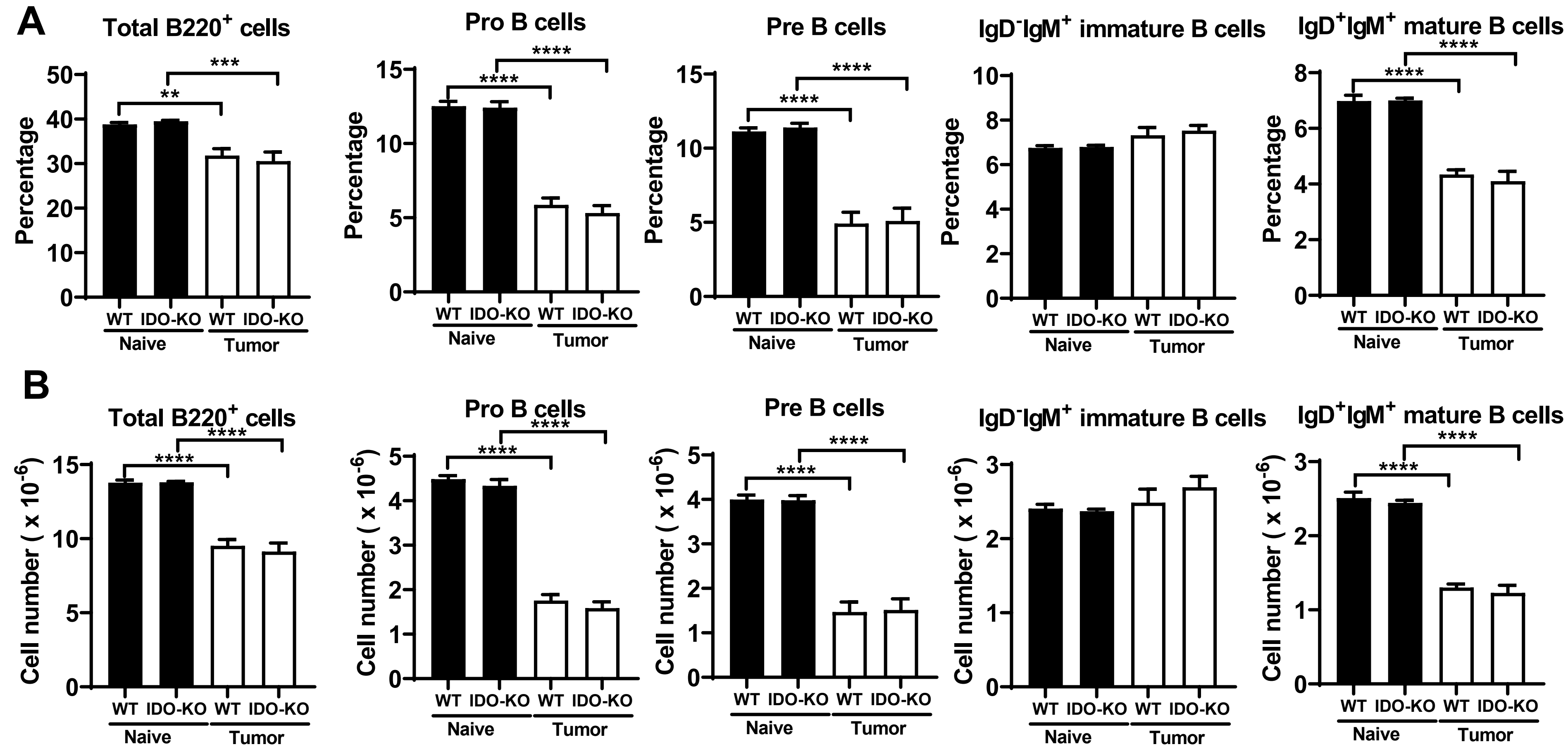

Supplementary Figure 9- IDO deficiency does not affect B cell development in BM. Percentages **(A)** and absolute numbers **(B)** of total B220$^+$, pro-, pre-, immature and mature B cells in BM of naïve or tumor-bearing WT and IDO KO mice on day 9 post-LLC i.c. challenge (n = 6 mice per group). Statistical significance was evaluated using one-way ANOVA with Tukey's multiple comparison testing. ** $P < 0.01$, *** $P < 0.001$, **** $P < 0.0001$.

Supplementary Figure 10

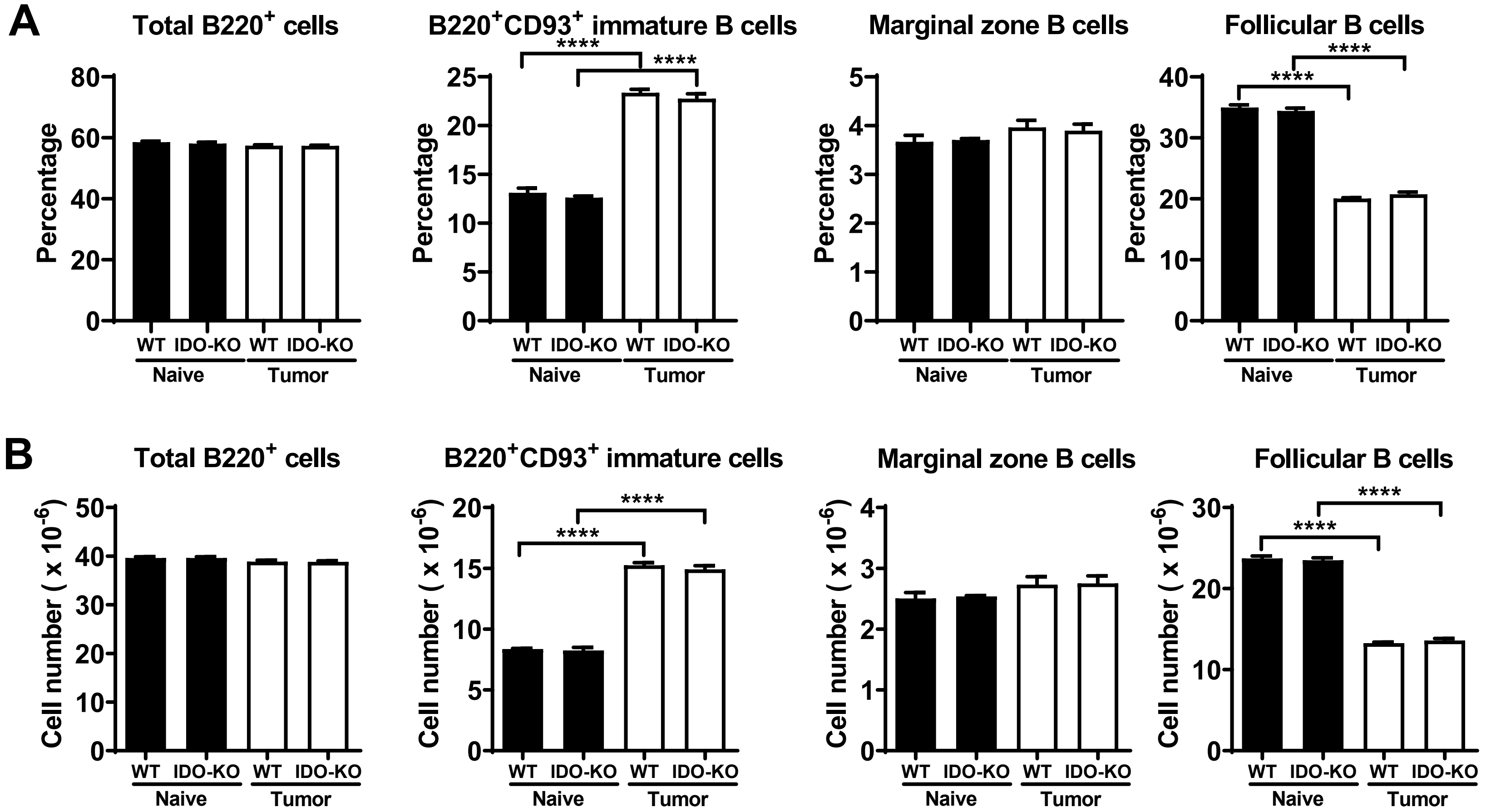

Supplementary Figure 10- IDO deficiency does not affect B cell development in spleen. Percentages and absolute numbers of total B220+, immature, marginal zone and follicular B cells in spleens of naïve or tumor-bearing WT and IDO KO mice on day 9 post-LLC i.c. challenge (n = 6 mice per group). Statistical significance was evaluated using one-way ANOVA with Tukey's multiple comparison testing. **** $P < 0.0001$.

Supplementary Figure 11

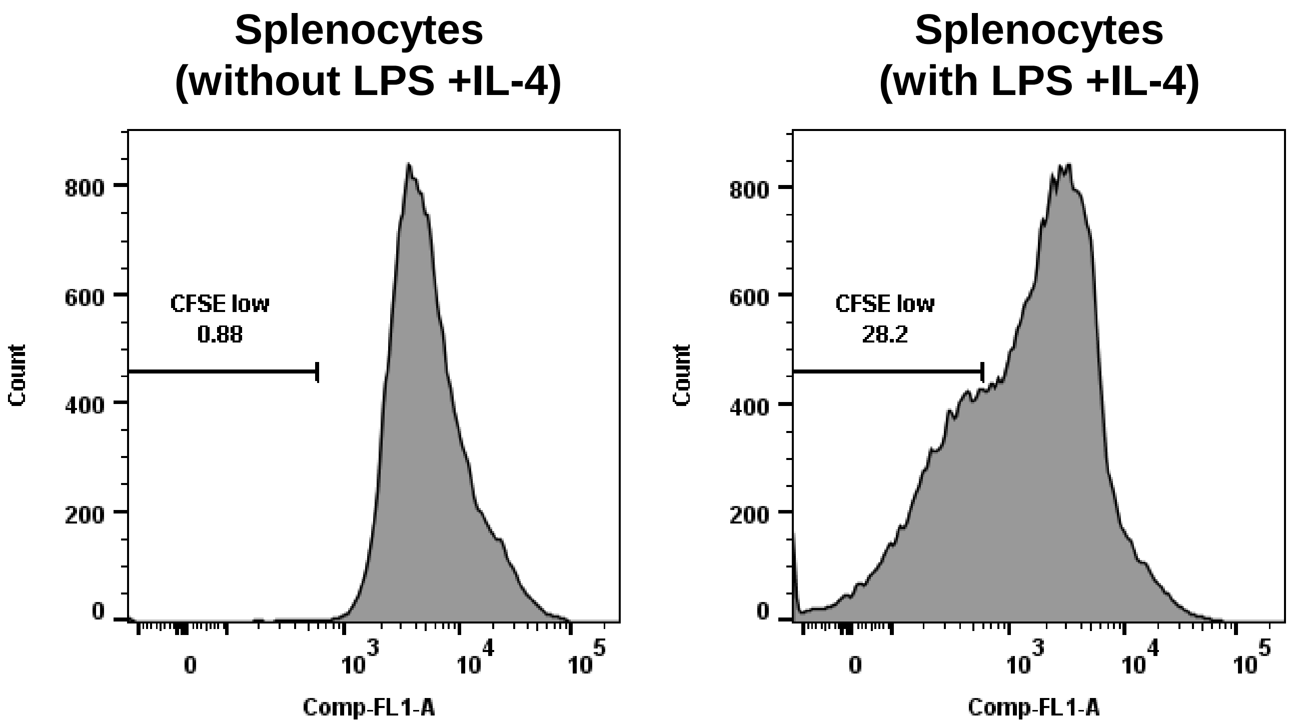

Supplementary Figure 11- Gating strategy to show CFSE low population
